# Nuclear Glycolytic Enzyme Enolase of *Toxoplasma gondii* Functions as a Transcriptional Regulator

**DOI:** 10.1371/journal.pone.0105820

**Published:** 2014-08-25

**Authors:** Thomas Mouveaux, Gabrielle Oria, Elisabeth Werkmeister, Christian Slomianny, Barbara A. Fox, David J. Bzik, Stanislas Tomavo

**Affiliations:** 1 Center for Infection and Immunity of Lille, CNRS UMR 8204, INSERM U 1019, Institut Pasteur de Lille, Université Lille Nord de France, Lille, France; 2 Laboratory of Cell Physiology, INSERM U 1003, Université Lille Nord de France, Villeneuve d'Ascq, France; 3 Department of Microbiology and Immunology, The Geisel School of Medicine at Dartmouth, Lebanon, New Hampshire, United States of America; University of Wisconsin Medical School, United States of America

## Abstract

Apicomplexan parasites including *Toxoplasma gondii* have complex life cycles within different hosts and their infectivity relies on their capacity to regulate gene expression. However, little is known about the nuclear factors that regulate gene expression in these pathogens. Here, we report that *T. gondii* enolase TgENO2 is targeted to the nucleus of actively replicating parasites, where it specifically binds to nuclear chromatin *in vivo*. Using a ChIP-Seq technique, we provide evidence for TgENO2 enrichment at the 5′ untranslated gene regions containing the putative promoters of 241 nuclear genes. Ectopic expression of HA-tagged TgENO1 or TgENO2 led to changes in transcript levels of numerous gene targets. Targeted disruption of TgENO1 gene results in a decrease in brain cyst burden of chronically infected mice and in changes in transcript levels of several nuclear genes. Complementation of this knockout mutant with ectopic TgENO1-HA fully restored normal transcript levels. Our findings reveal that enolase functions extend beyond glycolytic activity and include a direct role in coordinating gene regulation in *T. gondii*.

## Introduction

Apicomplexan parasites are important pathogens of humans and domestic animals that cause diseases with a widespread impact on global health. The life cycles of these obligate intracellular parasites are complex, involving multiple proliferative and non-growing stages that ensure successful parasite transmission. Pathogenesis, virulence, and disease severity are critically influenced by asexual stage growth rates that can lead to increased parasite biomass and significant tissue destruction and inflammation. *Toxoplasma gondii* is distinct from nearly all other members of the phylum Apicomplexa in its exceptionally large host range, which includes all warm-blooded animals. Although the advent of acquired immune deficiency syndrome (AIDS) has drawn attention to *T. gondii* as a serious opportunistic parasite, it has long been a major medical and veterinary problem responsible for causing abortion or congenital birth defects in both humans and livestock [1]. The infection is incurable because of the parasite's ability to differentiate from rapidly replicating tachyzoite stages into latent cysts containing the bradyzoite stages that are impervious to the host's immune system and current therapeutic drugs. *T. gondii* cysts and dormant bradyzoites persist in the brain of the infected host and play key roles in pathogenesis because they can convert to virulent tachyzoites in immune-compromised individuals with AIDS and transplant patients. This stage conversion is triggered by the host immune response and impairment of the immune system in HIV-infected individuals can lead to lethal toxoplasmic encephalitis. The ability of *T. gondii* to cycle between one parasitic stage and another, a process known as interconversion, is central to its pathogenesis. However, very little is known about the mechanisms involved in stage interconversion, and the key nuclear factors that control *T. gondii* differentiation remains to be discovered. Even more intriguing, completion of sequencing and annotation of the genomes of *T. gondii* and other apicomplexan parasites revealed a relatively low number of genes encoding transcription factors [2]–[7]. In contrast, the basal core transcriptional machinery and the protein-coding genes involved in nucleosome assembly and chromatin remodelling machinery were found to be well-conserved, leading to the proposal that gene regulation in *T. gondii* and other apicomplexan parasites is controlled mainly by epigenetic mechanisms [8]–[10]. However, the complexity of the parasite life cycle suggests that other nuclear factors are likely to be involved in both basic and stage-specific regulation of gene expression in the apicomplexan parasites. Recently, bioinformatics searches for DNA-binding domains have uncovered a family of proteins homologous to the plant transcription factor Apetala2, named ApiAP2 for apicomplexan AP2-like factor [11]–[17]. In *T. gondii*, two recent studies have described the transcriptional control of genes encoding key factors involved in translation (ribosomal proteins), host cell invasion, and modulators of immune responses (kinases, pseudo kinases, or ROP proteins of the apical secretory organelles named rhoptries) by nuclear ApiAP2 factors [14], [15]. Nevertheless, the balance between regulatory mechanisms and the relative roles of epigenetic and transcriptional machineries remain uncertain.

Our laboratory and others have previously established that *T. gondii* stage conversion is accompanied by the expression and nuclear localization of two stage-specific glycolytic enolases ENO1 (TgENO1) and ENO2 (TgENO2), also named 2-phospho-D-glycerate hydrolase (EC 4.2.1.11), that convert 2-phosphoglycerate to phosphoenolpyruvate in the glycolytic pathway of *T. gondii* [18]–[22]. TgENO1 is bradyzoite-specific enzyme only detected in the dormant encysted forms present in the brains of chronically infected mice while TgENO2 is exclusively expressed in the rapidly dividing and virulent tachyzoites [18]–[20]. During early intracellular proliferation and development, tachyzoites and bradyzoites exhibited very strong nuclear labelling for enolase, but in mature parasites this was markedly reduced to levels below those seen in the cytoplasm [21]. In addition, specific nuclear localization of TgENO2 has also been described in actively developing coccidian (both asexual and sexual) stages in a manner similar to the tachyzoites [21]. In mammalian cells, part of the enolase protein has been shown to function as a transcription factor that represses expression of the *c-myc* gene promoter [23], [24]. In the plant *Arabidopsis thaliana*, which has no bona fide *c-myc* homolog, cold-responsive gene transcription is controlled by a bi-functional enolase that binds to the promoter of the gene encoding the zinc finger protein STZ/ZAT10 [25]. In both animal and plant cells, binding of nuclear enolases to the TATA motif is required for the control of transcription regulation [23]–[25]. However, in contrast to mammalian and plant cells, a survey of the whole genome of *T. gondii* or other apicomplexan parasites reveals no functional TATA and CCAAT boxes in their gene promoters. Therefore, the functions associated with nuclear localization of *T. gondii* enolases remain to be elucidated.

Here, we report that TgENO1 and TgENO2 are preferentially targeted to the nucleus of intracellular and actively dividing *T. gondii*, where they regulate gene expression. Targeted disruption of the TgENO1 gene resulted in a decrease in brain cyst burden in chronically infected mice and in changes of transcript levels of several nuclear gene targets in bradyzoites. Complementation of this knockout mutant with ectopic TgENO1-HA restored these transcripts to normal levels. Our data show that TgENO1 and TgENO2 are nuclear factor that share the capacity for binding to putative gene promoters and to control gene expression during intracellular proliferation of *T. gondii*.

## Materials and Methods

### Parasite culture and nuclear extract preparation

Tachyzoites of the avirulent 76 K (type II) strain [19], [20] and the virulent *ΔKu80* (type I) strain [26], which was generously provided by Dr Vern Carruthers (University of Michigan, USA), were used in this study. These parasite strains were maintained *in vitro* by serial passage in confluent monolayers of human foreskin fibroblasts (HFF, from the American Type Culture Collection [ATCC]) grown in Dulbecco's modified Eagle's medium (Bio Whittaker) supplemented with 10% fetal calf serum (Gibco, BRL), 2 mM glutamine, and 0.05% gentamycin. Tachyzoites were allowed to grow until they lysed HFF cells spontaneously and were harvested by filtration through a glass wool column and a 3-µm pore filter. Encysted bradyzoites were purified from brains of chronically infected OlaHsd mice by pepsin digestion (0.05 mg/ml pepsin in 170 mM NaCl, 60 mM HCl) for 30 minutes at 37°C. Nuclear extracts were obtained from approximately 4×10^8^ purified tachyzoites of *T. gondii* 76K strain as previously described [22]. The quality of the nuclear extract was checked by Western blot analysis to confirm the absence of cytoplasmic contaminant (the glycolytic lactate dehydrogenase [27]) and enrichment of a *T. gondii* nuclear protein (TgDRE, a nuclear repair enzyme [28]).

### Experimental infection in mice and ethics statement

All animal experiments were performed following the guidelines of the Pasteur Institute Pasteur of Lille animal study board, which conforms to the **A**msterdam **P**rotocol on animal protection and welfare, and **D**irective 86/609/EEC on the **P**rotection of **A**nimals **U**sed for **E**xperimental and **O**ther **S**cientific **P**urposes, updated in the **C**ouncil of **E**urope's **A**ppendix A (http://conventions.coe.int/Treaty/EN/Treaties/PDF/123-Arev.pdf). The animal work also complied with the French law (n°87-848 dated 19-10-1987) and the **E**uropean **C**ommunities **A**mendment of **C**ruelty to **A**nimals **A**ct 1976. All animals were fed with regular diet and all procedures were in accordance with national regulations on animal experimentation and welfare authorized by the French Ministry of Agriculture and Veterinary committee (Permit number: 59-009145). The Pasteur Institute of Lille and the CNRS Committee on the Ethics of Animal Experiments specifically approved this study. Purified tachyzoites (10^3^ tachyzoites) from the parental 76K strain and the transgenic derivative ectopically expressing TgENO1-HA or TgENO2-HA were inoculated into groups of seven female 6- to 8-week-old CBA/J mice. For phenotypic studies of the TgENO1 knockout mutants, 5×10^2^ tachyzoites from the Pru*Δku80ΔTgeno1* mutant and parental Pru*Δku80* strain were purified as described above and inoculated into a group of six female 6- to 8-week-old Balb/C mice. ***In vivo*** cyst formation was determined by harvesting mouse brain 6–8 weeks after infection. Cysts were purified using Percoll gradients as described above and washed with PBS. The cyst wall was stained by FITC-labelled *dolichol biflorus* lectin and counted under observation by inverted phase microscopy.

### DNA manipulation and expression vector cloning

All primers used during this study and their purposes are described in Tables S1, S2 and S3. The open reading frames corresponding to TgENO1-HA and TgENO2-HA were amplified by polymerase chain reaction (PCR) using the forward and reverse primers indicated in Table S1. The forward primer contained a NsiI restriction site upstream of the coding sequence and the reverse primer contained a PacI restriction site downstream of the nucleotide sequence coding for the 12 amino acids of the HA epitope tag (CYPYDVPDYASL). PCR was performed as described previously [19] using the *T. gondii* 76K strain as a template, 50 pmol of each primer, and Pfu polymerase (Promega). The reaction conditions were 39 cycles of denaturation at 95°C for 1 minute, annealing at 66°C for 1 minute, and elongation at 72°C for 3 minutes, with a final elongation for 10 minutes at 72°C. PCR-amplified DNA fragments encoding TgENO1-HA and TgENO2-HA were double-digested with NsiI and PacI, gel-purified using the Gene clean spin kit (Q-Biogene) and cloned into the pSAG1-Bleo and pTUB5-Bleo vectors, respectively.

### *T. gondii* transfection and stable transformation

For ectopic expression of *T. gondii* enolases, 100 µg of the TgENO1-HA and TgENO2-HA DNA constructs was linearized by *Kpn*I, purified by phenol/chloroform extraction, resuspended in 100 µl cytomix and transfected into 76K strain tachyzoites using a BTX electroporation system as previously described [22]. Stable transformants were selected and cloned in the presence of 5 µg/ml bleomycin. Phenotypic studies of transgenic parasites over-expressing TgENO1-HA or TgENO2-HA were performed by plaque assays and direct staining and counting of intracellular parasites. Briefly, 10^3^ extracellular wild type or transgenic parasites were inoculated onto confluent HFF monolayers in 24-well plates for 2 hours at 37°C. Plaques were stained with crystal violet after 5–7 days in normal culture conditions. Experiments were repeated two or three times with triplicate wells. For the proliferation assay, 10^5^ parasites per well were incubated in a 24-well plate for 18, 24, or 36 hours. Fixation and staining was carried out using the RAL555 kit (RAL Diagnostics, France). The number of parasites per vacuole was counted in 15 fields per slide with a minimum of three slides per condition.

### Promoter studies

For promoter-driven expression, a plasmid containing the reporter luciferase gene and 787 bp of the 5′-untranslated region (UTR) containing the putative promoter of the TgMag1 gene (TGME49_070240) was constructed using the pair of primers described in Table S1. Two successive mutations of the parental TgMag1 plasmid were performed to generate *Δ*_1_ *Tg*Mag1 and *Δ*_2_ *Tg*Mag2 plasmids using the Quick-change Multi Site-Directed Mutagenesis Kit (Agilent). Briefly, the reaction mix was composed of 2.5 µl of 10× Quick-change Multi reaction buffer, 0.75 µl of Quick Solution, 50 ng of ds-DNA template, 100 ng of forward mutagenic primers, 100 ng of reverse mutagenic primers, 1 µl of dNTP mix, and 1 µl of Quick-Change Multi enzyme with double-distilled H_2_0 to a final volume of 25 µl. The mutagenesis reaction was performed by 29 cycles of 1 minute at 95°C, 1 minute at 55°C, and 2 minutes/kb of plasmid length at 65°C (13 minutes in this case). The PCR product was kept on ice for 2 minutes to cool the reaction and 1 µl of Dpn I restriction enzyme (10 U/µl) was added to digest the double-stranded DNA template. After digestion at 37°C for 1 hour, the mutated plasmid was used to transform *E. coli* and positive colonies were isolated.

### Generation of knockout mutants using ENO1-targeting plasmid and complementation

The type II bradyzoite-specific *ENO1* gene locus is defined by TGME49_068860 (Chromosome VIII, 6,242,505 to 6,244,976) in www.Toxodb.org (version 7.3). The plasmid *pΔENO1* was constructed by yeast recombinational cloning using previously described methods [29]. A 1,003-bp 5′ *ENO1* target flanking sequence amplified from type II Pru genomic DNA, the *HXGPRT* cDNA minigene cassette, and a 1,058-bp 3′ *ENO1* target flanking sequence amplified from type II Pru genomic DNA were fused into the yeast shuttle plasmid *pRS416*. The deletion was engineered to remove 366 bp of the *ENO1* 5′-UTR, the entire ENO1 protein coding region, and 283 bp of the *ENO1* 3′-UTR. The oligonucleotide primers used to construct *pΔENO1* and to validate targeted deletion of the *ENO1* gene are shown in Tables S2 and S3, respectively.

For complementation, the *ΔENO1* mutant was transfected with 30 µg of plasmid containing the coding region of ENO1 that was tagged with HA and flanked downstream by the heterologous promoter of the *T. gondii* sortilin (TgSORTLR) gene [30] and the 3′ UTR of the SAG1 gene. The linearized DNA was cleaned by ethanol precipitation and resuspended with 82 µl of Human T Cell Nucleofector solution plus 18 µl of buffer from the Amaxa Human T Cell Nucleofector Kit. A pellet containing 3×10^6^ *ΔENO1* mutant cells was resuspended in the DNA solution and transfection was performed using the Amaxa electroporation system and U33 program. After transfection, 10^5^ parasites were loaded onto a cover slide containing a confluent monolayer of HFF for the immunofluorescence assay (IFA) and the remaining parasites were used to infect a 25-cm^2^ flask containing confluent HFF cells, grown for 48 hours, and harvested for Western blot analysis. The intracellular parasites were cultured under tachyzoite growth (culture medium at pH 7) or bradyzoite growth (culture medium at pH 8) conditions.

### Electrophoretic mobility shift assays

Electrophoretic mobility shift assays (EMSA) were performed using a band shift assay kit (Thermo Scientific, France) according to the manufacturer's instructions. Gel retardation assays were performed with 27-bp biotinylated primers containing the TTTTTCTTCTC motif present in the TgMag1 promoter (probe A), the human TATA box (probe B), or an irrelevant control sequence (probe C) (Sigma, France). The biotinylated double-stranded oligonucleotides (20 fmol) were incubated for 20 min at room temperature with 1 µg of purified bacterial TgENO2 recombinant protein in binding buffer (10 mM Tris-HCl at pH 7.5, 50 mM NaCl and 0.5 mM DTT) containing 1 µg Poly(dI-dC):Poly(dI-dC) (Thermo Scientific, France), 0.01% NP40, and 10% glycerol. For competition experiments, a 100-fold excess of self or unrelated unlabeled double-stranded oligonucleotides was added to the binding reaction together with the recombinant TgENO2 protein and before addition of the labelled probe. Loading dye (25 mM Tris-HCl pH 7.5, 0.02% bromophenol blue, 0.02% xylene, and 4% glycerol) was added and the complexes were separated on 6% non-denaturing polyacrylamide gels in running buffer (7 mM Tris-HCl at pH 7.5, 3 mM NaC_2_H_2_O_2_ and 1 mM EDTA). The DNA was transferred onto a Nylon membrane and detected using the Light Shift Chemiluminescent EMSA Kit (Thermo Scientific, France).

### Western blots

Total protein extracts and pulled down proteins were resuspended in 25 µl Laemmli sample buffer, boiled, separated by SDS-PAGE, and transferred to Hybond ECL nitrocellulose (Amersham). Immunoblot analysis was performed using specific monoclonal anti-SAG1 (P30), anti-SAG4 (P36) or polyclonal anti-TgENO1 and anti-TgENO2 antibodies. The specificity of the rabbit polyclonal anti-TgENO1 and anti-TgENO2 antibodies has been previously described [19], [20]. The blots were incubated with peroxidase-conjugated secondary antibodies followed by chemiluminescence detection.

### Chromatin immunoprecipitation and high-throughput sequencing

Chromatin immunoprecipitation (ChIP) was performed as described previously [31] with slight modifications. Briefly, chromatin from intracellular parasites (wild type 76K or ENO2-HA strain) grown in HFF cells (three 150-cm2 flasks) was cross-linked for 10 minutes with 1% formaldehyde at room temperature and purified as described [31]. After cross-linking, the extracts were sonicated to yield chromatin fragments of 500–1,000 bp. Immunoprecipitations were performed using polyclonal anti-ENO1 and anti-ENO2 antibodies and monoclonal or polyclonal anti-HA antibodies (Invitrogen). The immunoprecipitate was incubated at 4°C overnight and washed as described [31]. DNA was subjected to proteinase K digestion for 2 hours and purified using the Qiagen PCR purification kit. Pre-immune sera were used as a negative control. The primers used for PCR are listed in Table S1. ChIP PCR products were electrophoresed on agarose gels, stained with ethidium bromide, and photographed using a UV-light scanner.

For ChIP-Seq, chromatin was immunoprecipitated by anti-ENO2 antibody as above and amplified using a GenomePlex Amplification of ChIP DNA kit (Sigma-Aldrich). The amplified ChIP products were electrophoresed on agarose gels and stained with ethidium bromide. DNA fragments with a length of 500 bp or less were purified and processed for high-throughput sequencing (Genoscreen, Pasteur Institute of Lille). The GsFLX bead adaptors and specific tag (MID) were introduced to the flanking 5′ and 3′ end of each purified DNA sample according to the manufacturer's instructions. The ChIP-Seq GsFLX libraries were analyzed on a Bio analyzer 2100 using Agilent RNA 6000 Pico methods and quantified using Quant-iT TM RiboGreen (Invitrogen). Equimolar amounts of the libraries were mixed, fixed on beads, and amplified using the GS FLX Titanium emPCR Kit (454 Life Sciences, Roche Diagnostics). The beads were purified, enriched, counted using Beckman Coulter Z1, and deposited on the GS FLX Titanium Pico Titer Plate (454 Life Sciences, Roche Diagnostics). The pyro sequencing reaction was performed using a GS FLX Titanium Sequencing Kit and Genome Sequencer FLX Instrument (454 Life Sciences, Roche Diagnostics). For bioinformatics analyses, the GSMapper software v 2.3 (Roche) was used to align reads for each sample using the updated *T. gondii* ME49 genome databases downloaded from http://www.toxodb.org. Only sequences of a minimum overlapping length of 40 nucleotides with at least 90% identity were considered for further analyses. The ChIP-Seq data specific to TgENO2 or TgENO2-HA were subtracted from non-specific sequences obtained with the pre-immune sera used as negative control and data collected for specific sequences immunoprecipitated by anti-ENO2 and anti-HA antibodies were grouped into four sub-groups of associated contigs. Signal Map software was used to schematically represent the number of reads per contig and their corresponding positions, which allowed visualization of putative gene promoter occupancy by ENO2 for all chromosomes of *T. gondii*.

### Quantitative real-time PCR and RT-PCR

Anti-ENO2 or anti-HA chromatin immunoprecipitates were subjected to qPCR to validate genes identified by ChIP-Seq or to quantify the level of chromatin that was specifically immunoprecipitated. For quantitative reverse transcriptase PCR (qRT-PCR), tachyzoites of wild-type parasites and parasites that over-expressed TgENO1-HA or TgENO2-HA were purified from infected HFF cells and RNA from 10^8^ tachyzoites was reverse transcribed for 1 hour at 42°C in a buffer containing 1 M oligo (dT)_18_ primer, 2 mM dNTPs, 40 U rRNasin (Promega) and 25 U AMV reverse transcriptase (Roche) for subsequent PCR amplification using the primers listed in Table S1. The T. gondii β-tubulin housekeeping gene was used as a negative control. We confirmed that each primer pair amplified a single product, identified as a single band in an acrylamide gel and as a single peak within the qPCR dissociation curve. The primer pairs displayed amplification efficiency greater than 90%. Quantitative PCR was performed with the Maxima TM SYBR Green qPCR Master Mix Kit (Fermentas) using the Mx3005P TM real-time PCR System (Stratagene). ROX Solution was used as a passive reference for all analyses and the qPCR was repeated three times, each in duplicate. Gene expression in TgENO1 and TgENO2 over-expressing parasites was represented as fold expression relative to wild-type after normalization.

### Immunofluorescence assay and confocal imaging

The immunofluorescence assay (IFA) was performed as described previously [32]. Briefly, 2×10^5^ intracellular tachyzoites were fixed with 4% paraformaldehyde in PBS for 15 minutes on ice, washed twice with PBS, and dried on Teflon slides. Intracellular parasites were permeabilized with 0.1% Triton X-100 in PBS containing 0.1% glycine for 10 minutes at room temperature. Samples were blocked with 3% BSA in the same buffer and then primary antibodies were added in the same buffer and incubated for 1 hour at 37°C. Secondary antibody coupled to Alexa-488 or Alexa-633 (Molecular Probes; diluted 1∶1000) was added together with DAPI to stain the nucleus. The samples were examined with a Zeiss Axioplan microscope or by confocal imaging with a LSM710 microscope (Zeiss) and a Plan Apochromat objective (Plan-Apochromat 63x/1.40 Oil DIC M27, Zeiss) as previously described [31]. Quantification of TgENO2 signal in the nucleus and cytoplasm of the intracellular parasites was performed using 8-bit images (256 grey levels) that were acquired sequentially and averaged four times. Size was adapted to the optimal resolution that could be obtained by the confocal setup (0.13 µm per pixel). UV excitation (laser diode 405 nm) enabled imaging of the DAPI signal of parasite nuclei (blue image corresponding to spectral range 410–495 nm) and excitation at 561 nm allowed imaging of the TgENO2 signal (red fluorescence corresponding to spectral ranges 565–700 nm). Image analysis was performed using ImageJ (NIH) software. A short macro enabled us to automatically determine the relative proportion of TgENO2 signal in the nucleus versus the cytoplasmic signal. The cytoplasm of parasite was delimited by fluorescent background staining of anti-ENO2 while the DAPI enabled imaging parasite nuclei as indicated above. For each image, regions of interest (ROIs) delimiting the nucleus and cytoplasm were determined and the integrated intensity was calculated for each ROI as described (30). The proportion of signal contained in the nuclei corresponds to 100× (Nucleus intensity)/(Nucleus + Cytoplasm intensity).

### Statistical analysis

Statistical differences between groups of mice used in this study were evaluated by the Student's t-test. Mann-Whitney test was used for analysis of intracellular growth of TgENO1-HA and TgENO2-HA *versus* wild type tachyzoites after colorimetric staining and microscopic observation and for mouse survival curves.

## Results

### TgENO2 is dynamically shuttled from the cytoplasm to the nucleus of actively dividing *T. gondii*

**Figure 1:**
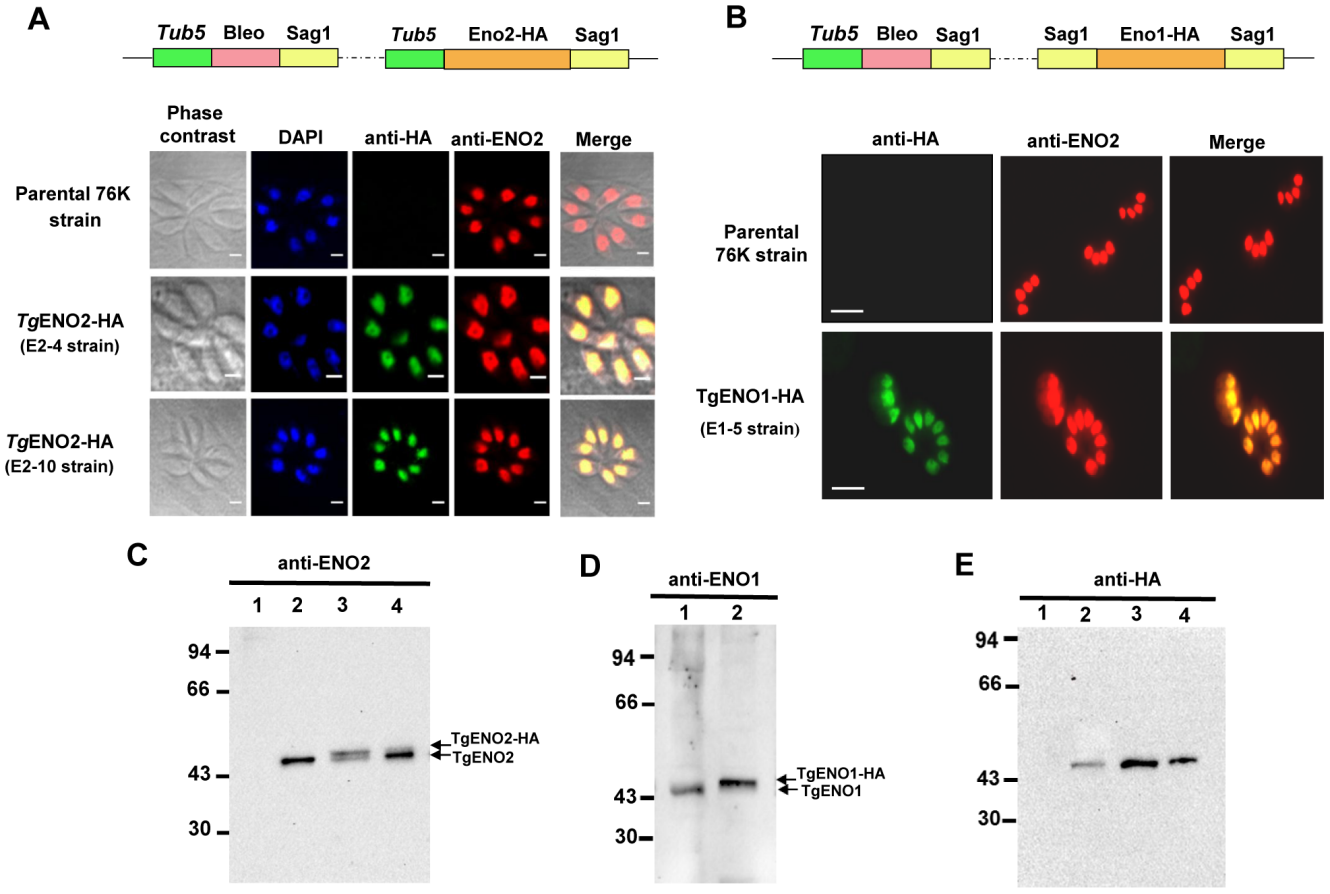
Ectopic expression of TgENO2-HA and TgENO1-HA in wild type *T. gondii* 76K tachyzoites confirms predominant nuclear enolase localization. A) Schematic representation of the vector used for stable ectopic expression of TgENO2-HA in parasite clones. Expression of TgENO2-HA protein driven by the heterologous tubulin gene promoters was demonstrated by co-localization of fluorescent images using anti-HA and anti-ENO2 antibodies. Similar to endogenous TgENO2 protein, ectopic TgENO2-HA was exclusively localized in the nucleus of two stable clones (E2-4 and E2-10) selected after bleomycin treatment. B) Schematic representation of the vector used for stable ectopic expression of TgENO1-HA in parasite clones. Expression of TgENO1-HA protein driven by heterologous tubulin gene promoters was demonstrated by co-localization of fluorescent images obtained using anti-HA and anti-ENO1 antibodies. Similar to endogenous ENO1 protein, TgENO1-HA protein was exclusively localized in the nucleus of one stable clone (E1-5) selected after bleomycin treatment. C) Western blots of transgenic and ectopically expressed TgENO2-HA protein. Total protein extracts from E2-4 and E2-10 tachyzoites were loaded in lanes 2 and 3 and probed with anti-ENO2. Lane 2 contained total protein from wild-type 76K *T. gondii* tachyzoites. Lane 1 corresponds to total protein extract from encysted bradyzoites isolated from the brain of mice chronically infected with wild type *T. gondii* 76K strain. C) Western blots of transgenic and ectopically expressed TgENO1-HA protein. Total SDS-extracted proteins from E2-4 and E2-10 transgenic tachyzoites were loaded in lanes 3 and 4 and probed with anti-ENO2 antibodies. Lane 2 contained total SDS-extracted proteins from wild-type 76 K *T. gondii* tachyzoites and lane 1 corresponds to total SDS-extracted proteins from encysted bradyzoites isolated from the brains of mice chronically infected with wild type *T. gondii* 76K strain. D) Western blot analysis of transgenic and ectopically expressed TgENO1-HA protein. Total SDS-extracted proteins from the E1-5 strain was loaded in lane 2 and probed with anti-ENO1 antibodies. Lane 1 corresponds to total-SDS extracted proteins from encysted bradyzoites as described above. E) Western blots of total SDS-extracted proteins from E1-5, E2-4 and E2-10 tachyzoites were loaded in lanes 2, 3 and 4 and probed with anti-HA antibodies. Lane 1 contained total SDS-extracted proteins from wild-type 76 K *T. gondii* tachyzoites.

We previously reported that rabbit polyclonal anti-TgENO1 and anti-TgENO2 antibodies that are highly specific to ENO1 and ENO2 protein, respectively, and do not show cross-reactivity to their mammalian counterparts detect these two glycolytic enolase isoenzymes in the nuclei of bradyzoites and tachyzoites, respectively [19], [20]. To rule out the possibility of cross-reaction with other unrelated parasitic nuclear proteins, we expressed ectopic Tg-ENO2 tagged with influenza hemagglutinin (HA) in the tachyzoites of 76K (type II) strain under the control of a strong tubulin gene promoter (Fig. 1A). Immunofluorescence assays (IFA) confirmed that the ectopic TgENO2-HA recognized by the rat polyclonal anti-HA antibody perfectly co-localized with endogenous TgENO2 and that the signal was strongly detected in the nuclei of intracellular parasites (Fig. 1A).

Because of the limitations of obtaining sufficient numbers of encysted bradyzoites from brains of chronically infected mice, we engineered transgenic tachyzoites that ectopically expressed TgENO1-HA under the control of the promoter from the major surface antigen 1 (*SAG1*) gene. Figure 1B shows a representative positive clone (E1-5) in which ectopic TgENO1-HA co-localized with nuclear endogenous TgENO2, similar to ectopic TgENO2-HA shown in Figure 1A. The anti-HA fluorescent signals were perfectly superimposed with anti-ENO2 staining (red signals), demonstrating that both transgenic bradyzoite-specific TgENO1-HA and TgENO2 were properly targeted to the nuclei of actively dividing tachyzoites (merged pictures). We confirmed the expression of both ectopic TgENO2-HA and TgENO1-HA by Western blots using polyclonal antibodies specific to HA (Fig. 1E, lanes 2-4), anti-ENO2 (Fig. 1C, lanes 3 and 4) and TgENO1-HA (Fig. 1D, lane 2). The endogenous TgENO2 protein in tachyzoites (Fig. 1C, lane 2) and TgENO1 protein of encysted bradyzoites isolated from the brains of chronically infected mice (Fig. 1D, lane 1) were used to monitor the protein levels of these positive controls.

### The level of nuclear enolases increased in actively dividing tachyzoites of *T. gondii*

**Figure 2:**
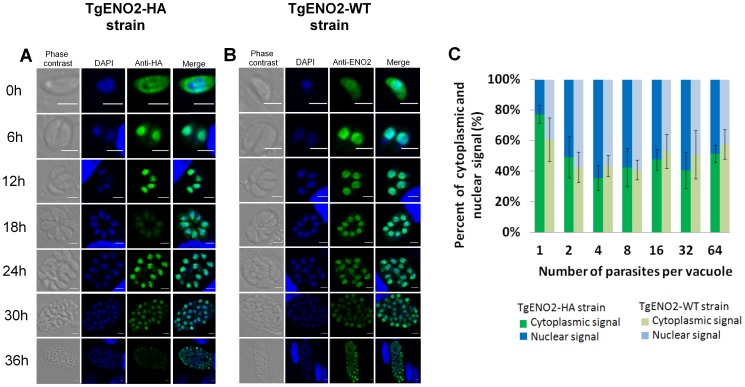
Expression of ectopic TgENO2-HA and native TgENO2 proteins is predominantly nuclear and increases with intracellular replication of *T. gondii*. A) Kinetics of nuclear accumulation of ectopic TgENO2-HA protein in transgenic tachyzoites at different time points (0, 6, 12, 18, 24, 30, and 36 h post-infection) during the intracellular division cycle. Intracellular dividing transgenic tachyzoites were fixed and stained with rabbit polyclonal anti-HA and DAPI followed by confocal imaging. Scale bars, 5 µm. B) Kinetics of nuclear accumulation of native TgENO2 protein in wild type *T. gondii* tachyzoites at different time points (0, 6, 12, 18, 24, 30, and 36 h post-infection) during the intracellular division cycle. Intracellular dividing transgenic tachyzoites were fixed and stained with rabbit polyclonal anti-ENO2 antibodies and DAPI followed by confocal imaging. Scale bars, 5 µm. C) Quantification of cytoplasmic and nuclear levels of ectopic TgENO2-HA and native TgENO2 in intracellular dividing tachyzoites of *T. gondii*. Experiments were repeated three times (n = 3, P<0.001). Quantifications were performed on at least 8–10 independent intracellular vacuoles using ImageJ software and bioinformatics tools as described in Materials and Methods.

We examined the subcellular localization of TgENO2-HA and its endogenous counterpart in intracellular tachyzoites at 0, 6, 12, 18, 24, 30, or 36 hours post-infection using immunofluorescence confocal microscopy (Fig. 2A and 2B). These kinetic studies revealed that the proportion of ectopic TgENO2-HA and endogenous TgENO2 detected in the nuclei of intracellular tachyzoites increased from 20% to 60% during the 12-h period of intracellular growth, reaching 70% by 32 h post-invasion (Fig. 2C). The increased nuclear fluorescence of TgENO2-HA and TgENO2 correlated with the increase in the number of intracellular tachyzoites from 1 to 32 daughter parasites (Fig. 2C). Conversely, the level of cytoplasmic TgENO2 in the tachyzoites decreased to 20–30% (Fig. 2C). We conclude that a substantial proportion of ectopic and endogenous enolases are dynamically targeted to the nuclei of actively replicating tachyzoites. These data also suggest that the glycolytic enolase of *T. gondii* might have novel nuclear functions that are distinct from its classic role as a cytoplasmic enzyme required for energy production.

### Genome-wide TgENO2 occupancy of gene promoters defined by ChIP-Seq

We next investigated the interactions of TgENO2 with 5′-UTR gene sequences that might correspond to putative gene promoters *in vivo* using chromatin immunoprecipitation followed by high-throughput sequencing (ChIP-Seq). Intracellular actively dividing tachyzoites of wild-type *T. gondii* 76K strain or transgenic TgENO2-HA parasites were fixed by formaldehyde and released from host cells. After chromosome fragmentation by sonication, the chromatin was immunoprecipitated using specific anti-TgENO2 or anti-HA antibodies, or with pre-immune or non-relevant anti-ROP1 sera as negative controls. The immunoprecipitates were subjected to high-throughput sequencing and bioinformatics analyses using genome data from http://www.toxodb.org. After comparison of sequences and removal of common genes that were targeted by both pre-immune and specific anti-TgENO2 and anti-HA sera, the 5′-UTRs corresponding to the putative promoters of 241 genes that were commonly pulled down by both antibodies were selected (Table S4). The gene promoters pulled down were expressed in both tachyzoite and bradyzoite stages, suggesting that there is no clear link between genes targeted by nuclear enolase and their stage-specific expression (Table S4). In addition, these antibodies also bound to coding regions of the same genes or other gene targets (Table S5). We have no obvious explanations of the binding of nuclear TgENO2 to coding regions of these target genes identified by ChIP-Seq. We cannot rule out the possibility that binding to gene coding regions may also account for transcription regulation. However, we do not further investigate the role of TgENO2 binding to coding regions because this issue is beyond the scope of this study. Therefore, we focused on TgENO2 produced hits that were localized to promoter regions. Among the gene promoters identified were mostly 44% (107) of encoded hypothetical proteins, 4% encoded metabolic enzymes, 5% encoded translation factors, 5% encoded cytoskeleton, trafficking, and transporter proteins, and 12% encoded other enzymes (Table S4). Interestingly, ChIP-Seq also identified 5 genes corresponding to the novel plant-like AP2 transcription factor AP2 that have been demonstrated to regulate transcription in apicomplexan parasites [13]–[17]. We conclude that *T. gondii* enolase may be important in nuclear functions linked to the transcription and translation of genes involved in parasite growth and intracellular development. Surprisingly, very few genes coding surface proteins, or microneme and rhoptry components were identified with the stringency set at reads of a minimum of 40 bp.

**Figure 3:**
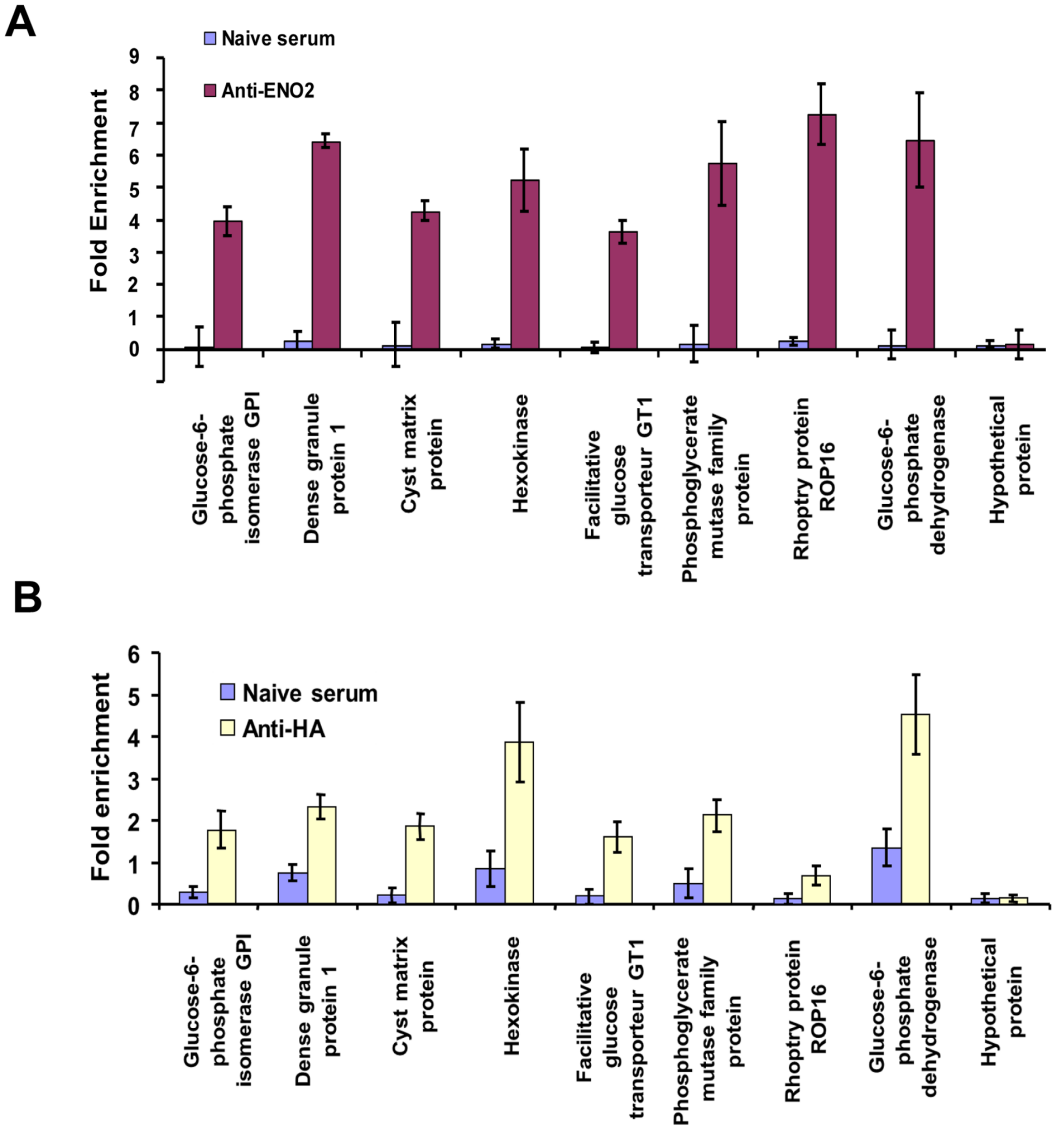
Validation of native TgENO2 and transgenic TgENO2-HA protein bound to several putative gene promoters. A) Quantitative real-time PCR analysis of chromatin immunoprecipitates from three independent experiments (n = 3, P<0.0001) demonstrates specific binding of nuclear TgENO2 *in vivo* to eight selected genes identified by ChIP-Seq (see Table S4). A gene encoding a hypothetical protein that was absent from the gene hits (TgME49_ 0022080) was used as a negative control. B) Quantitative real-time PCR analysis of chromatin immunoprecipitates from three independent experiments (n = 3, P<0.0001) demonstrates specific binding of nuclear transgenic TgENO2-HA *in vivo* to eight selected genes identified by ChIP-Seq (see Table S4). The TgME49_ 0022080 gene was used as a negative control.

Quantitative real-time PCR analysis was used to demonstrate the specificity of chromatin immunoprecipitates from wild-type and TgENO2-HA strains pulled down with anti-TgENO2 (Fig. 3A) and anti-HA (Fig. 3B) antibodies, respectively. We confirmed that eight genes (glucose-6-phosphate isomerase, dense granule protein 1 (GRA1), cyst matrix protein 1 (TgMag1), hexokinase, glucose transporter putative, phosphoglycerate mutase, rhoptry protein 16 (ROP16), and glucose-6-phosphate dehydrogenase) were significantly and specifically targeted by nuclear TgENO2 *in vivo* using anti-ENO2 (Fig. 3A) and anti-HA antibodies (Fig. 3B). As a negative control, a gene (TgME49_ 0022080) encoding a hypothetical protein that was not present in the list of nuclear TgENO2 target genes (Table S4) was not amplified (Fig. 3A and 3B), confirming specific binding of nuclear TgENO2 *in vivo* on these eight selected genes. These experiments were performed three times from two independent ChIP experiments with reproducible and statistically significant results (P<0.0001). We also pulled down the 5′-UTRs corresponding to ROP16, TgENO1, and TgENO2 with a stringency of nucleotide length less than 40 bases (approximately 20–30 bp), suggesting that the number of gene hits of nuclear TgENO2 may actually be higher than the 241 genes shown in Table S4. Pre-immune (naïve) serum did not bind to these eight genes. We conclude that TgENO2 is targeted to the nuclei of intracellular dividing tachyzoites of *T. gondii* where it specifically binds to the promoter regions of numerous genes.

### Evidence for specific *T. gondii* enolase-DNA interactions

**Figure 4:**
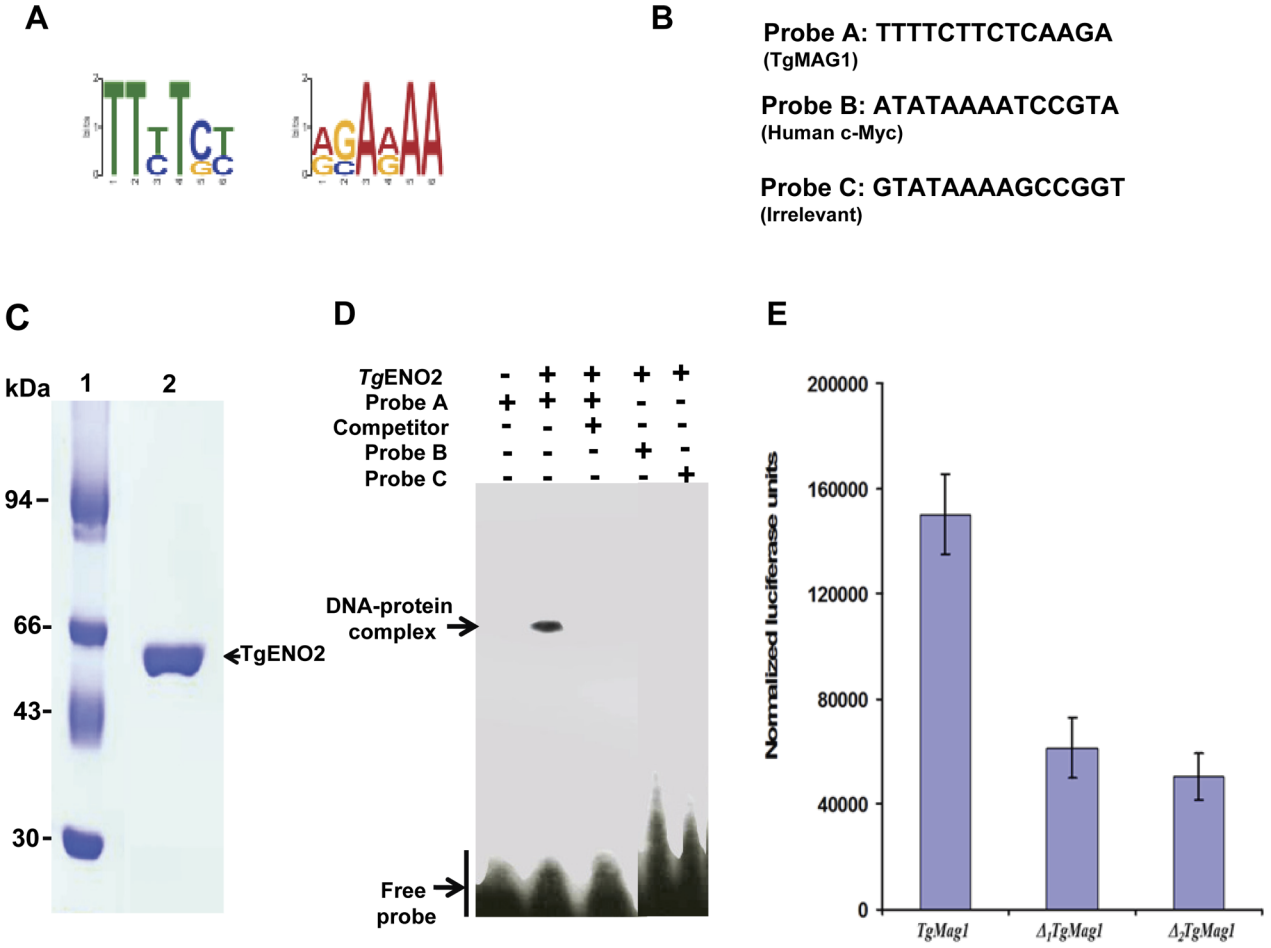
Role of the TTTTCT motif in specific TgENO2-DNA interactions and promoter activity. A) The TTTTCT motif was identified in the putative gene promoters targeted by nuclear TgENO2 using ChIP-Seq (Table S4) and the MEME bioinformatics tool (a motif-based sequence analysis tool). B) Nucleotide sequences of probes corresponding to the TTTTCT motif in the promoter of TgMag1 gene, the TATA box from human c-Myc gene, and a non-relevant motif used as a negative control. C) Expression and purification of recombinant TgENO2 fused to His-Tag. D) Electrophoretic band shift assays using recombinant TgENO2 incubated with or without the probes described in panel A. The unlabeled competitor was present at 100-fold excess. E) The GCTAGC motif is required for efficient transcription of the TgMag1 gene. The putative promoter of TgMag1, corresponding to a 787-bp region upstream of the start codon, was subjected to site-directed mutagenesis resulting in sequential disruption of the single TTTCT motif within the TTTTTCTTCTC motif of TgMag1 to *ATCGA*TCTC (*Δ*_1_ *Tg*Mag1) and then to *ATCGAGCGC* (*Δ*_2_ *Tg*MAg2). These two mutant promoters and the wild-type promoter were cloned upstream of a reporter luciferase construct and assayed for their ability to drive transcription. The transcriptional potential of mutated promoters was measured as firefly luciferase activity normalized to activity of a vector encoding *Renilla* luciferase. These experiments have been performed three times (n = 3, p<0.001).

**Figure 5:**
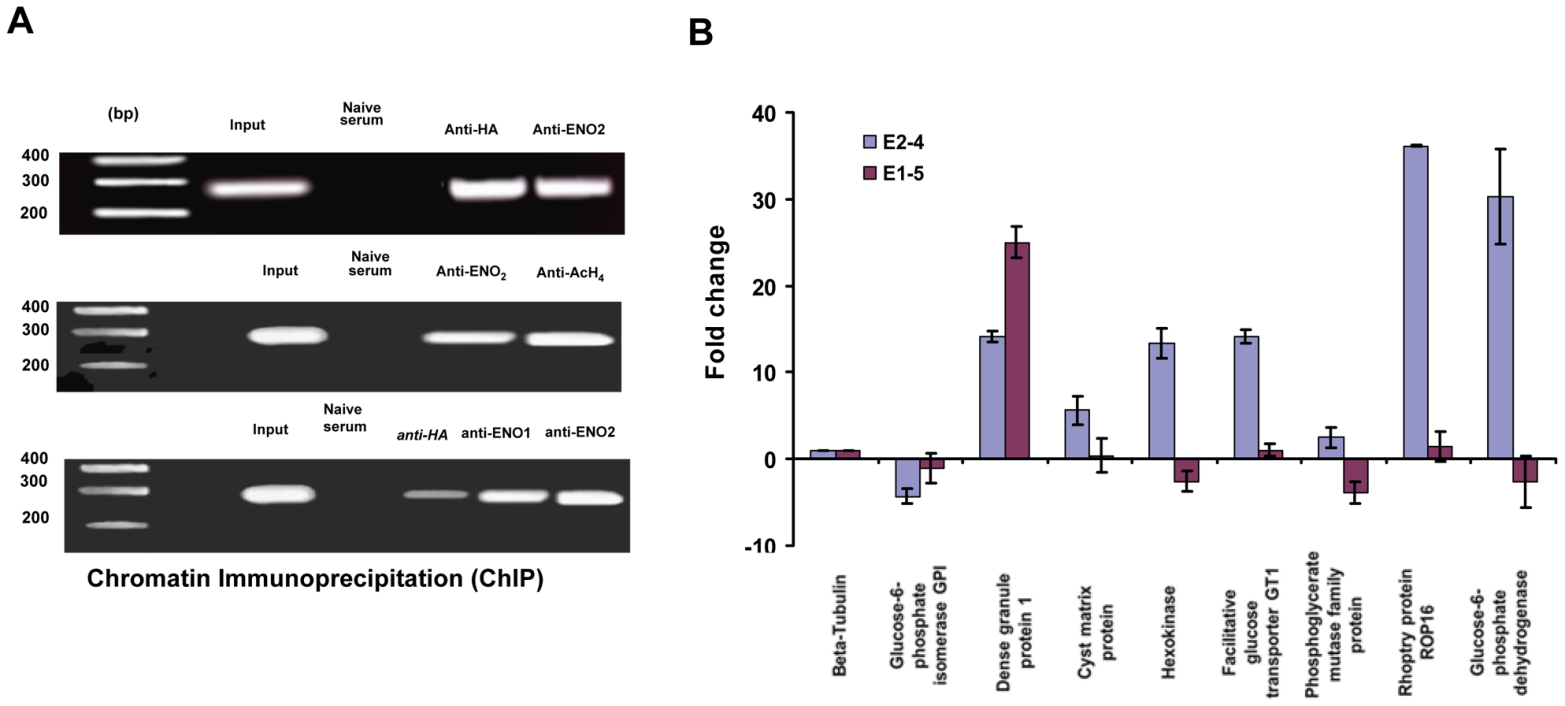
Nuclear enolases bind to promoters of several genes including their own and modulate transcriptional expression of several gene targets. A) The upper panel shows comparative chromatin immunoprecipitation (ChIP) from intracellular tachyzoites of a strain that ectopically overexpressed TgENO2-HA (E2-4) using polyclonal anti-HA and anti-ENO2 antibodies. No chromatin was immunoprecipitated with a non-relevant non-immune serum used as negative control. The middle panel shows chromatin immunoprecipitation (ChIP) of intracellular parasites that ectopically express TgENO2-HA (strain E2-4) with polyclonal antibodies specific to TgENO2 or acetyl histone H4, the epigenetic mark of an active promoter. The lower panel shows ChIP of parasites that ectopically express TgENO1-HA parasites (strain E1-5) with polyclonal antibodies specific to HA, TgENO1, and TgENO2. B) Changes in gene expression were confirmed by qRT-PCR analysis in the intracellular tachyzoites over-expressing TgENO2-HA and TgENO1-HA, compared with the beta-tubulin housekeeping gene and the expression of these genes in wild-type parasites. These experiments were repeated twice with triplicate samples and identical results were obtained n = 3, (P<0.001).

Searches for a conserved motif common to the gene promoters targeted by nuclear TgENO2 using the bioinformatics MEME tool identified the motif TTTTCT (Fig. 4A), which is present at least once in 238 out of the 241 putative promoters targeted by TgENO2 and listed in Table S4. The TTTTCT motif was detected in the 2,500-bp sequence upstream of the start codon (ATG) of 99% of the target genes of TgENO2. However, this motif is also present in the promoter of many other genes in *T. gondii* genome that were not pulled down by TgENO2 in addition to its presence in coding regions, suggesting that this motif alone may not be sufficient for the efficient and specific binding to gene promoters *in vivo*. Nevertheless, we established that the TTTTCT motif is directly involved in DNA-protein interactions *in vitro* using bacterial recombinant TgENO2 protein, which was purified to homogeneity (Fig. 4C) and tested for its binding to this motif designed from the 787-bp putative promoter of *TgMAG1*, one of the target genes identified in Table S4. It should be mentioned that TgMAG1 also called cyst matrix antigen is not a bradyzoite specific protein as was originally described [33] but was later shown to be expressed by both tachyzoites and bradyzoites [34]. Figure 4D showed that recombinant TgENO2 protein specifically interacted with the TTTTCT motif. In contrast, TgENO2 protein did not bind to a c-Myc motif from animal or plant [23]–[25] that contains a canonical TATA box (probe B), or to the unrelated probe C (Fig. 5B). Thus, we conclude that nuclear enolases of *T. gondii* could possibly bind to DNA motif present in the promoter regions of parasite genes, which may allow multi-complex nuclear factors to control gene expression in intracellular proliferating tachyzoites.

### The TTTTC motif is involved in promoter activation

Given that TgENO2 clearly targets the TTTTC motif in many putative gene promoters and the bacterially purified recombinant protein also binds specifically to this motif, it seems likely that this motif is responsible, at least in part, for regulating gene expression. To address this hypothesis, the putative promoter region of TgMag1 was fused to firefly luciferase and the double TTTTC-like motif (TTTTTCTTCTC, see TgMag1 in Figure S1) present in the promoter was first mutated to *ATCGA*TCTC (*Δ*_1_ *Tg*Mag1, Figure S2), followed by a second mutation to *ATCGAGCGC* (*Δ*_2_ *Tg*MAg2, Figure S3). A dual luciferase assay was carried out after transient transfection with constructs containing wild-type or mutated promoters of TgMag1 gene in addition to vector encoding *Renilla* luciferase for standardization. The *ATCGA*TCTC mutation in TgMag1 promoter (*Δ*_1_ *Tg*Mag1) resulted in a 60% decrease in luciferase expression compared with wild-type (Fig. 4E). The combination of both mutations in *Δ*_2_ *Tg*Mag2 (*ATCGAGCGC*) resulted in a 70% reduction in luciferase expression compared with wild-type (Fig. 4E). We conclude that deletion of the TTTTC motif in TgMag1 promoter has a significant negative effect on its activity *in vivo*.

### TgENO1 and TgENO2 bind to promoters *in vivo* and modulate gene expression

Next, we showed that both endogenous nuclear TgENO2 or ectopically expressed TgENO2-HA and TgENO1-HA enolases bound to the active gene promoter of TgENO2 in rapidly replicating tachyzoites using ChIP assays (Fig. 5A). We also demonstrated that ectopic TgENO1-HA, which is illegitimately expressed in the transgenic tachyzoites, bound specifically to the *TgENO2* gene promoter *in vivo* similar to the binding of nuclear TgENO2-HA (Fig. 5A). We conclude that nuclear enolases can bind to their own gene promoters and probably modulate gene activation and repression in T. gondii. Additionally, we showed that the RNA profiles of these genes described above were significantly modulated by ectopic nuclear TgENO1-HA and TgENO2-HA (Fig. 5B). The quantitative RT-PCR data shown in Figure 5B reveal that four out of the eight genes tested were negatively regulated in intracellular E1-5 tachyzoites expressing TgENO1-HA (Fig. 5B, red columns). In contrast, six out of eight genes were positively regulated in the intracellular E2-4 tachyzoites expressing TgENO2-HA (Fig. 5B, blue columns). The mRNA level of the housekeeping β-tubulin gene was unchanged, as expected. We conclude that nuclear enolases are likely involved in the transcriptional control of numerous genes through binding to their promoter regions. These data suggest that nuclear TgENO2 and TgENO1 associate with chromatin, leading to either up-regulation or down-regulation of gene expression.

### Targeted disruption of the bradyzoite-specific TgENO1 gene

To gain more insight into the biological functions of nuclear enolases during infection, we aimed to knock out the TgENO1 and TgENO2 genes. Despite several attempts, we failed to obtain targeted deletion of the tachyzoite-specific TgENO2 in intracellular tachyzoites using direct or inducible gene disruption strategies. This failure seems to suggest that TgENO2 is essential for intracellular parasite growth, as might be expected, considering its nuclear regulatory roles in addition to its function in glycolysis and ATP production. Alternatively, it may reflect the weaker strength of the conditional promoter compared with the TgENO2 promoter, or the *Tgeno2* locus may be refractory to double homologous recombination. Nevertheless, we did successfully target the *Tgeno1* locus by double homologous recombination for deletion of the bradyzoite-specific TgENO1 gene in intracellular tachyzoites (Figure S4). A PmeI-linearized pΔENO1 targeting plasmid was transfected into *T. gondii* strain Pru*Δku80Δhxgprt* that exhibits highly enhanced homologous recombination [29], and transfected parasites were selected in mycophenolic acid (MPA). MPA-resistant clones were isolated and the genotype of the clones was evaluated by PCR (Fig. S4A and S4B). In PCR1, the EXR primer was paired with the CXF primer (Table S2) to verify the presence of the 5′ target flank in all clones and the parental strain. PCR2 was used to identify clones with a deleted *ENO1* gene by the absence of PCR product (clones 1–12, Fig. S4B) compared with the parental strain (lane 13, Fig. S4B). To verify targeted integration of *HXGPRT* into the deleted *ENO1* locus, we used PCR3 and PCR4 to respectively verify 5′ and 3′ integration of the HXGPRT selectable marker in the entire ENO1 knockout (Fig. S4B). For further phenotypic studies, we selected one of these positive *T. gondii* clones and confirmed specific deletion of the entire open reading frame of TgENO1 gene (Fig. S4C, see primers in Table S3).

### Targeted deletion of TgENO1 reduces cyst burden in the brain of chronically infected mice

**Figure 6:**
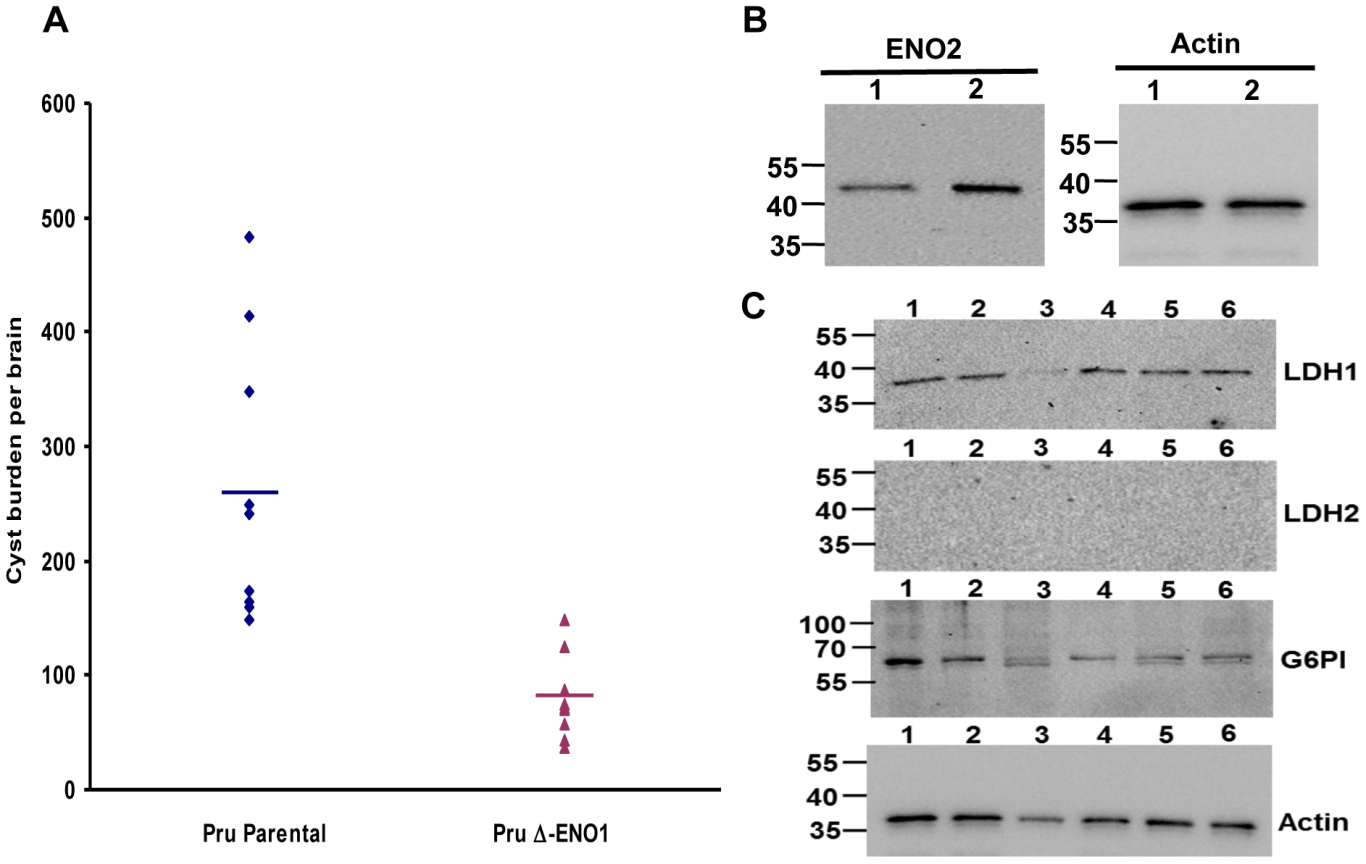
Targeted deletion of TgENO1 reduces cyst burden in the brain of chronically infected mice. A) The total number of cysts per brain of mice infected with 5×10^2^ tachyzoites from the Pru*Δku80ΔTgeno1* mutant or parental Pru*Δku80* was counted after staining with FITC-labeled *dolichol biflorus* lectin. A group of nine mice was used for each experiment, and the experiment was repeated twice with similar results (n = 2, P<0.001). Cyst burden (total number of cysts per brain) of the Pru*Δku80ΔTgeno1* mutant was significantly lower than that of the parental Pru*Δku80* strain. B) Western blots of total SDS-extracted proteins from knockout Pru*Δku80ΔTgeno1* mutants (lane 1) and parental Pru*Δku80* tachyzoites (lane 2). Left panel was probed with the polyclonal anti-ENO2 antibodies while the right panel was stained with the monoclonal anti-actin antibodies. C) Western blots of mutants and wild type parasites. Lane 1, total SDS-protein extracts from wild type 76K tachyzoites. Lane 2, total SDS-extracted proteins from transgenic E1-5 tachyzoites. Lane 3, total SDS-extracted proteins from transgenic E2-4. Lane 4, total SDS-extracted proteins from transgenic E2-10. Lane 5, total SDS-extracted proteins from parental Pru*Δku80* tachyzoites. Lane 6, total SDS-extracted proteins from knock-out Pru*Δku80ΔTgeno1* mutants. Blots were stained with polyclonal antibodies specific to LDH1, LDH2, G6PI and monoclonal antibodies specific to actin. The numbers on the left indicate molecular markers in kilodaltons.

We consistently noticed a severe decrease in the number of brain tissue cysts in mice infected with TgENO1 knockout mutants compared with those infected with parental parasites (Fig. 6A). Specifically, at 4 weeks post-infection, the average of brain tissue cyst burden in mice inoculated with parental Pru*Δku80* was about 300 cysts per brain compared with 50 cysts per brain in mice infected with the knockout Pru*Δku80ΔTgeno1* mutants. These experiments were independently performed three times using different preparations of freshly harvested extracellular tachzyoites with identical results. Furthermore, we observed a significant over-expression of the tachyzoite-specific TgENO2 in the knockout Pru*Δku80ΔTgeno1* mutants as shown by Western blots (Fig. 6B, left panel, compare lane 2 to lane 1). As compared to the housekeeping actin used for to verify loading of equal protein amounts (Fig. 6B, right panel), it appears that TgENO2 protein level in Pru*Δku80ΔTgeno1* mutants is approximately two-fold higher (Fig. 6B, left panel, lane 2) than that of the parental Pru*Δku80* parasites (Fig. 6B, left panel, lane 1). In contrast, the levels of other tachyzoite-specific glycolytic enzymes such as lactate dehydrogenase LDH1, glucose 6-phosphate isomerase (G6PI) were unchanged in *Δku80ΔTgeno1* mutants *versus* the parental Pru*Δku80* parasites (Fig. 6C, lanes 5 and 6). In addition, the knock-out of TgENO1 does not alter the expression of the bradyzoite-specific LDH2 enzyme (Fig. 6C, LDH2 panel, lanes 5 and 6). Again, the housekeeping actin was used as a loading control (Fig. 6C, Actin panel). In addition, we also showed that the ectopic expression of TgENO1-HA and TgENO2-HA in the E1-4, E2-4 and E2-10 strains used for ChIP-sequencing and qPCR does not have any obvious consequences in the protein levels of other glycolytic enzymes such as LDH1, LDH2 and G6-PI (Fig. 6C, lanes 2, 3 and 4). Taken together, these data indicate that the intracellular Pru*Δku80ΔTgeno1* mutants and the ectopic TgENO1 and TgENO2 parasite over-expressers grew normally as tachyzoites under our experimental culture conditions.

### Targeted deletion of TgENO1 directly influenced transcriptional regulation

**Figure 7:**
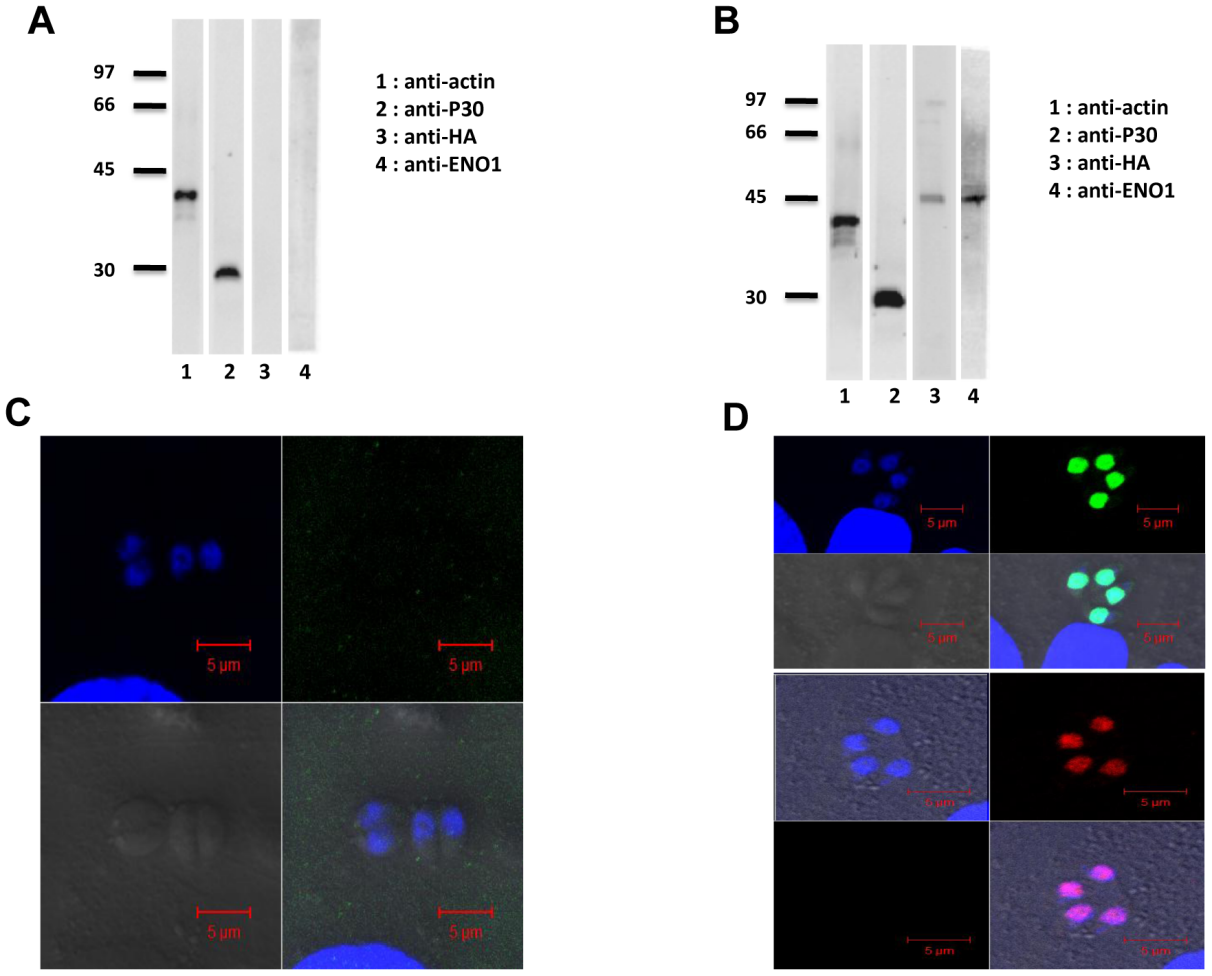
Complementation of Pru*Δku80ΔTgeno1* knockout mutant by ectopic expression of TgENO1-HA under tachyzoite culture conditions. A) Western blot analysis of Pru*Δku80ΔTgeno1* mutants using total SDS-extracted antigens from approximately 2×10^6^ tachyzoites and probed with monoclonal antibodies specific to *T. gondii* actin (a housekeeping protein, lane 1), tachyzoite-specific major surface protein 1 or SAG1 (anti-P30, lane 2), the influenza hemagglutinin (HA)-tag (anti-HA, lane 3) and TgENO1 (lane 4). B) Western blot analysis of total SDS-extracted antigens from approximately 2×10^6^ intracellular tachyzoites from complemented Pru*Δku80ΔTgeno1* mutants and probed with antibodies specific to *T. gondii* actin (lane 1), tachyzoite-specific major surface protein 1 or SAG1 (anti-P30, lane 2), the influenza hemagglutinin (HA)-tag (anti-HA, lane 3) and TgENO1 (lane 4). C) Confocal microscopy of intracellular tachyzoites of Pru*Δku80ΔTgeno1* mutants that were grown in confluent HFF cells and stained with polyclonal anti-HA antibodies. The panels show nuclei stained by DAPI (upper left panel), IFA (upper right panel), phase contrast (lower left panel), and merged image (lower right panel). Scale bars are 5 µm. D) Confocal microscopy of intracellular tachyzoites from Pru*Δku80ΔTgeno1* mutants that were complemented with TgENO1-HA plasmid and grown in confluent HFF cells. The first four upper panels show staining with polyclonal anti-HA antibodies (green). The second four lower panels show double staining with FITC-labeled *dolichol biflorus* lectin (cyst wall marker, green) and polyclonal anti-HA antibodies (red). The panels show nuclei stained with DAPI (upper left panel, blue), IFA (upper right panel, green or red), phase contrast (lower left panel), and merged image (lower right panel). Scale bars are 5 µm.

**Figure 8:**
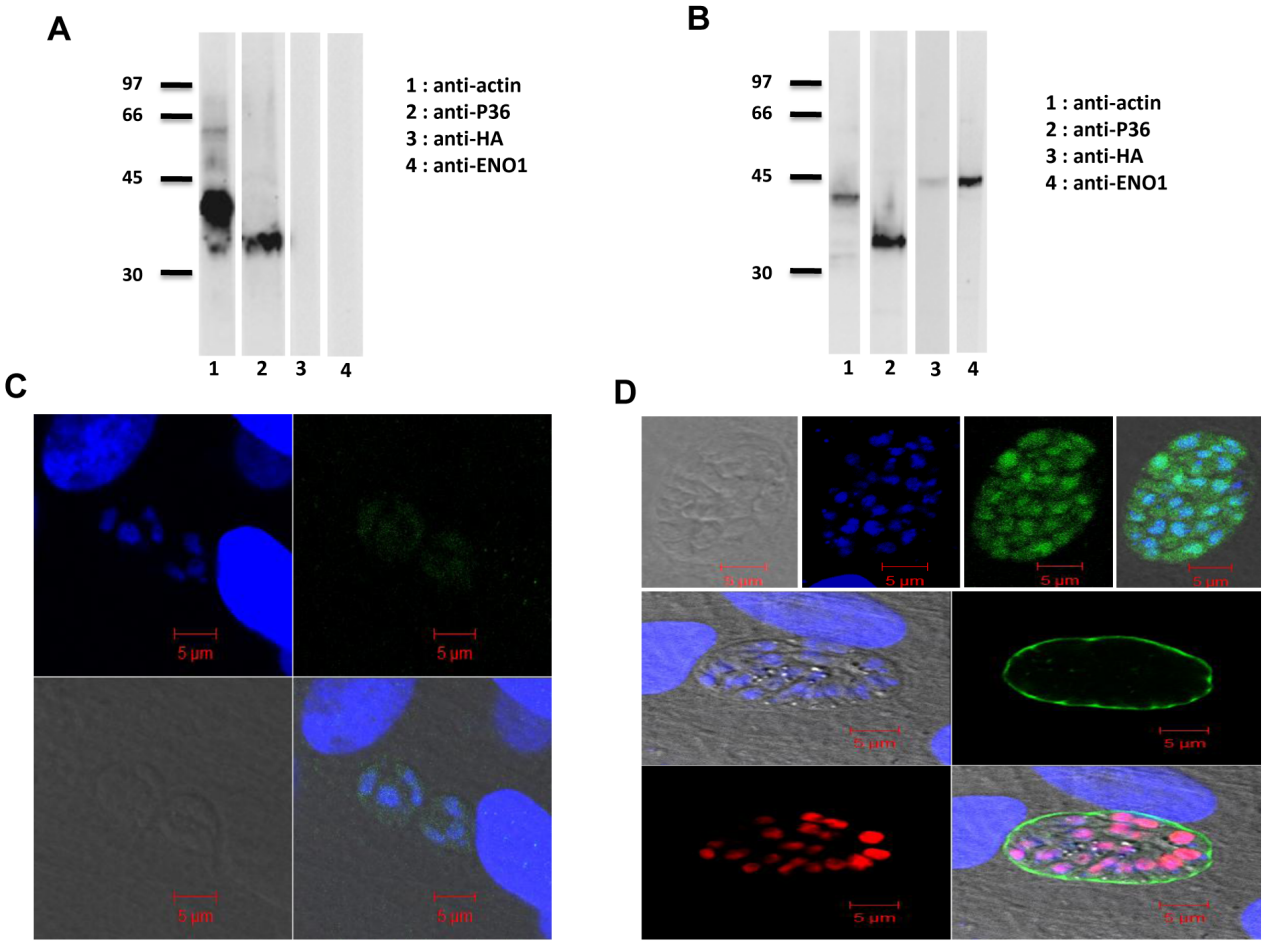
Complementation of Pru*Δku80ΔTgeno1* mutants by ectopic expression of TgENO1-HA and growth under bradyzoite culture conditions. A) Western blot analysis of Pru*Δku80ΔTgeno1* mutants using total SDS-extracted antigens from *in vitro*-induced intracellular bradyzoites and probed with monoclonal antibodies specific to *T. gondii* actin (lane 1), bradyzoite-specific major surface protein 1 (anti-P36, lane 2), the influenza hemagglutinin (HA)-tag (anti-HA, lane 3) and TgENO1 (lane 4). B) Western blot analysis of complemented Pru*Δku80ΔTgeno1* mutants using total SDS-extracted antigens from *in vitro*-induced intracellular bradyzoites and probed with monoclonal antibodies specific to *T. gondii* actin (lane 1), bradyzoite-specific major surface protein (anti-P36, lane 2), the influenza hemagglutinin (HA)-tag (anti-HA, lane 3) and TgENO1 (lane 4). C) Confocal microscopy of *in vitro*-induced intracellular bradyzoites of Pru*Δku80ΔTgeno1* mutants that were grown in confluent HFF cells and stained with polyclonal anti-HA antibodies. The panels show nuclei stained with DAPI (upper left panel), IFA (upper right panel), phase contrast (lower left panel), and merged images (lower right panel). Scale bars are 5 µm. D) Confocal microscopy of *in vitro*-induced intracellular bradyzoites of complemented Pru*Δku80ΔTgeno1* mutants that were grown in confluent HFF cells and stained with anti-HA antibodies (upper panels show phase contrast, DAPI, IFA, and merged images). Complemented Pru*Δku80ΔTgeno1* mutants were grown in confluent HFF cells before double staining with FITC-labeled *dolichol biflorus* lectin (cyst wall marker, green) and polyclonal anti-HA antibodies (red). The nuclei were stained by DAPI (left middle panel, blue). IFA reveals staining of the cyst wall (right middle panel, green) and ectopic TgENO1-HA protein expressed in the complemented Pru*Δku80ΔTgeno1* mutants was stained by anti-HA antibodies (lower left panel, red). Merged images are shown in the lower right panel. Scale bars are 5 µm.

To validate the phenotypic consequences of the Pru*Δku80ΔTgeno1* knockout, we complemented this mutant with a second plasmid carrying the TgENO1 gene that ectopically expressed the enzyme. We verified that TgENO1 protein was not expressed in the tachyzoites of the Pru*Δku80ΔTgeno1* knockout mutants using Western blot analysis, monoclonal anti-P30, which is directed to tachyzoite-specific surface antigen 1 (SAG1), and anti-TgENO1 antibodies (Fig. 7A) and confocal microscopy (Fig. 7C) whereas the complemented mutants clearly expressed ectopic TgENO1-HA protein (Fig. 7B and 7D). The complemented mutants also expressed ectopic TgENO1-HA under *in vitro* bradyzoite-induced conditions (increased alkaline pH), as shown by Western blot analysis using anti-HA (Fig. 8B, lane 3), anti-ENO1 antibodies and monoclonal anti-P36, which is directed to bradyzoite-specific surface antigen SAG4 [37] (Fig. 8B, lane 4). Confocal microscopy showed that TgENO1-HA protein was localized to the nuclei of the alkaline pH-induced bradyzoites (Fig. 8D), with positive staining of the cyst wall by the lectin WGA marker [35], [36]. As expected, no endogenous TgENO1 protein was detected in the Pru*Δku80ΔTgeno1* mutant transfected with empty plasmid under alkaline pH bradyzoite-induced conditions (Fig. 8A and 8C).

Having confirmed that complementation of the Pru*Δku80ΔTgeno1* knockout mutant with an ectopically expressing vector resulted in the expression of TgENO1-HA that was localized to the nuclei of tachyzoites and *in vitro*-induced bradyzoites, we could directly compare the effect of the absence or presence of nuclear TgENO1 protein on the transcriptional regulation of tachyzoite and bradyzoites of *T. gondii*.

**Figure 9:**
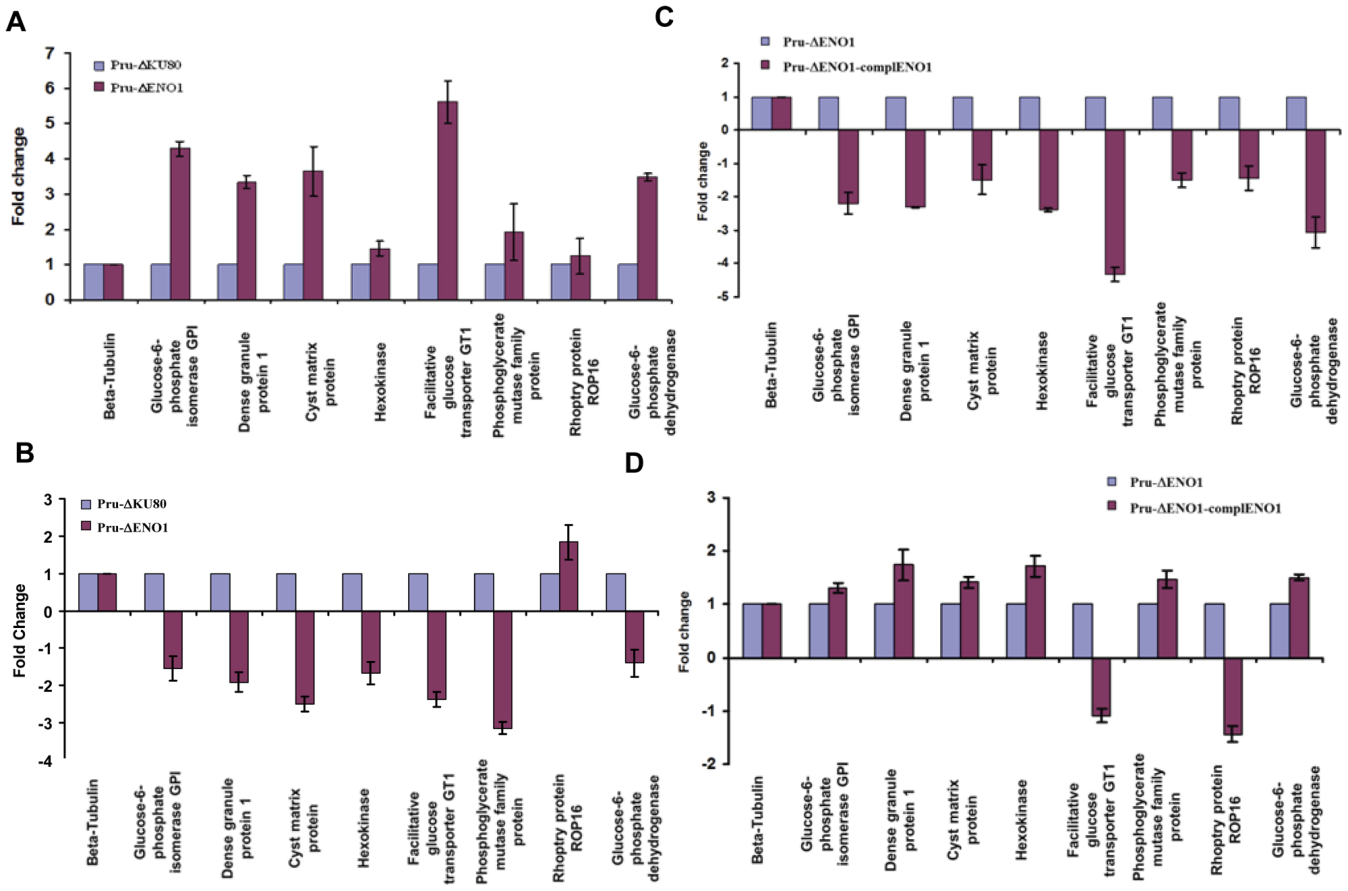
Targeted deletion of TgENO1 and complementation of the knockout demonstrate that nuclear enolase modulates gene transcript levels. A) Total RNA extracted from intracellular tachyzoites of Pru*Δku80ΔTgeno1* mutants or parental Pru*Δku80* grown in normal culture conditions was reverse transcribed and used for quantitative RT-PCR. The eight enolase target genes selected by ChIP-Seq and beta tubulin, a housekeeping gene, were analyzed. B) Total RNA was extracted from intracellular tachyzoites of the Pru*Δku80ΔTgeno1* mutant or parental Pru*Δku80* after bradyzoite interconversion *in vitro* using alkaline (pH 8) stress culture conditions. After reverse transcription, the cDNA was subjected to quantitative RT-PCR analysis of the eight enolase target genes selected by ChIP-Seq and beta tubulin. C) Total RNA extracted from intracellular tachyzoites of Pru*Δku80ΔTgeno1* mutants complemented with TgENO1 plasmid or parental Pru*Δku80* parasites grown in normal culture conditions was reverse transcribed and used for quantitative RT-PCR analysis of the eight enolase target genes and beta tubulin. D) Total RNA was extracted from intracellular tachyzoites from Pru*Δku80ΔTgeno1* mutant complemented with TgENO1 plasmid or parental Pru*Δku80* parasites after bradyzoite interconversion *in vitro* using alkaline (pH 8) stress culture conditions. After reverse transcription, the cDNA was subjected to quantitative RT-PCR analysis of the eight enolase target genes selected by ChIP-Seq and beta tubulin. These experiments were performed at least three times with identical results (n = 3, P<0.0001).

We compared transcript levels under experimental conditions that support the intracellular growth of tachyzoites (normal pH 7) or stress-induced bradyzoite differentiation (alkaline pH 8.2), as an *in vitro* experimental model of bradyzoite and cyst formation, by qRT-PCR. We found that expression of the eight nuclear genes targeted by the nuclear TgENO2 was differentially modulated 2- to 5-fold between intracellular tachyzoites of Pru*Δku80ΔTgeno1* mutants and parental Pru*Δku80* (Fig. 9A). Specifically, the transcript levels of five genes were increased in the mutant and those of three genes were unchanged (Fig. 9A). In contrast, transcript levels of seven of these eight genes were consistently decreased in *in vitro*-induced bradyzoites from Pru*Δku80ΔTgeno1* mutants relative to parental Pru*Δku80* (Fig. 9B). We noticed that complementation of Pru*Δku80ΔTgeno1* mutants by ectopic expression of TgENO1-HA did not significantly affect the decrease in transcript levels of these eight target genes in the tachyzoites (Fig. 9C) whereas the normal transcript levels of most gene targets (5 out 7) were restored in *in vitro*-induced bradyzoites, as expected for a functional complementation (Fig. 9D). We did not compare the production of cysts in the brains of chronically infected mice because we were unable to obtain stable complemented parasite lines despite several attempts. In addition, the low number of cysts obtained in mice brains also limited the quantity of RNA that could be isolated for qRT-PCR. Nevertheless, our data indicate that lack of TgENO1 has a direct impact on gene regulation and influences transcript levels during the *in vitro* interconversion between the rapidly replicating tachyzoite and the dormant bradyzoites of *T. gondii*.

## Discussion

In the present study we aimed to decipher novel functions of stage-specific glycolytic enolases in *T. gondii* that display dual cytoplasmic and nuclear localization, but are considerably enriched in the nuclei of actively dividing intracellular parasites. It is somewhat intriguing that no nuclear localization sequence (NLS) is present in *T. gondii* enolases, although the presence of a NLS has not been described in any of the human, plant, or parasite enolases that are efficiently transported into the nucleus for repression of gene transcription [23]–[25]. We cannot rule out the possibility that enolases enter the nucleus through interaction with a partner or chaperone that contains a functional NLS. Additionally, the size of enolase is close to the cut off for the nuclear pore, and it is possible that an NLS is not required but the protein might be trapped in one compartment or another under the appropriate conditions. To date, the mechanism by which enolases and other glycolytic enzymes are transported into the nucleus remains unknown and represents an interesting issue that is beyond the scope of our present investigation. Here, we focused on elucidating the novel nuclear functions of *T. gondii* enolases as an important step towards a better understanding of gene regulation in *T. gondii*. We have previously shown that silencing of tachyzoite enolase 2 (TgENO2) using double-strand RNA inhibition strategies alters the nuclear targeting of bradyzoite enolase 1 (TgENO1) [38]. These observations suggest that concomitant expression of both isoenzymes in the nuclei of intermediate parasitic forms at the early stages of interconversion might play a role in their nuclear targeting and in the regulation of genes involved in parasite differentiation and cyst formation [36]. Here, we demonstrate that *T. gondii* enolase binds specifically to approximately 241 putative gene promoters *in vivo*, which represents about 3% of the total gene content in the parasite genome. We provide further evidence that this binding positively or negatively influences gene expression in *T. gondii*.

For many years, glycolytic enzymes have been considered to be housekeeping cytoplasmic proteins. However, recent studies have provided evidence that some glycolytic enzymes are multifaceted and perform multiple functions, rather than just being simple components of the glycolytic pathway [39]. For example, glycolytic enzymes have been shown to have additional functions in transcriptional regulation (hexokinase-2, lactate dehydrogenase, glyceraldehyde-3-phosphate dehydrogenase or GAPD), stimulation of cell motility (glucose-6-phosphate isomerase), and the regulation of apoptosis (glucokinase and GAPD) [39]. It is well established that enolase is a multifunctional protein that is involved in gene transcription in other eukaryotes [23]–[25], [39] including the protozoan parasite *Entamoeba histolytica* [40]. Although no non-glycolytic functions have been described for *T. gondii* lactate dehydrogenases (LDH1 and LDH2), which are exclusively localized to the cytosol [21], [27], *T. gondii* aldolase is a key component of the actin-myosin motor essential for parasite gliding and host cell invasion [41], [42]. The results of this study support the notion that *T. gondii* nuclear enolases also have a non-glycolytic function through binding to gene promoters *in vivo*, leading to a decrease or increase in transcript levels of numerous genes. In mammals and plants, nuclear enolases modulate gene regulation through direct binding to a TATA box in their target gene promoters [23]–[25]. Interestingly, we found that *T. gondii* enolases are very different from their counterparts in higher eukaryotes because they do not bind to the TATA motif of mammalian and plant gene promoters. This is consistent with the fact that no functional TATA motif has been shown to be involved in transcription regulation of *T. gondii* [43], [44] or other apicomplexan parasites. Moreover, *T. gondii* enolases also have striking differences from higher eukaryote counterparts with regard to their plant-like structural peculiarities [19], [20]. We did, however, identify a putative common TTTTCT motif within the promoter sequences of all *T. gondii* enolase target genes identified by ChIP-Seq. Using gel retardation assays; we further demonstrated that bacterial recombinant *T. gondii* ENO1 and ENO2 proteins specifically bound to this TTTTCT motif. Additionally, deletion of this motif TTTCT motif from the putative promoter of gene encoding the cyst matrix antigen, also known as TgMag1 that is present in both bradyzoites and tachyzoites, significantly decreased the promoter activity *in vivo*, suggesting that binding of nuclear enolases to this motif may be involved in gene regulation. We cannot rule out the possibility that the nuclear functions of TgENO2 may also involve epigenetic mechanisms. Future identification of other nuclear partners that specifically interact with TgENO2 will advance our understanding of how nuclear enolases control gene expression in *T. gondii*.

To date, only a few nuclear factors have been characterized in *T. gondii* and other apicomplexan parasites [6], [7], [11]–[17], [31], [45] and little is known about specific *cis*-elements and nuclear factors that regulate gene expression in these parasites [11]–[17], [43], [44]. We demonstrate that ectopic expression of *T. gondii* enolases or targeted disruption of the *TgENO1* gene directly impacts transcript levels. Moreover, knockout of the TgENO1 gene results in a significant decrease in the cyst burden in the brains of chronically infected mice, suggesting that nuclear TgENO1 may be important for controlling expression of genes that are required for proper and efficient bradyzoite differentiation and cyst formation. We cannot rule out that the bradyzoites formed by the TgENO1 KO mutants may also have other defects. The limited number of brain tissue cysts and bradyzoites obtained from the KO mutants did not allow us to further examine the ultrastructure of these mutants by electron microscopy. Nevertheless, our inability to generate knockout mutant of the tachyzoite-specific TgENO2 despite several attempts and various strategies including the conditional KO method indicates that *T. gondii* enolases may be key nuclear factors, and at least one isoform of enolase should always be present in the nuclei of the parasites to ensure their intracellular growth and development. However, we observed no significant difference between the *in vitro* intracellular replication rates of the Pru*Δku80ΔTgeno1* ENO1 knockout and the parental Pru*Δku80* strains. In addition, both KO and parental strains are able to mount acute toxoplasmosis in mice. The presence of TgENO1 protein in the nuclei of the complemented Pru*Δku80ΔTgeno1* mutant and its restorative effect on transcriptional regulation *in vitro* strongly suggests that nuclear targeting of these glycolytic enzymes may represent the key sensor that regulates gene transcription during stage interconversion. However, we cannot exclude the possibility that the heterologous TgSORTLR gene promoter [30] used to complement the TgENO1 KO mutants may induce an effect on the timing of activity or target occupancy by an already active TgENO1 protein in the tachyzoite. Nevertheless, our complementation with this ectopic TgENO1-HA successfully restored the changes provoked by the deletion of endogenous TgENO1gene, as expected.

In conclusion, our data reveal the existence of new nuclear regulatory functions for nuclear enolases in *T. gondii*. We hypothesize that links may be established between metabolic sensors and transcription through *T. gondii* enolases or other enzymes that participate in cell metabolism. Our previous findings indicated that TgENO1 and TgENO2 display distinct enzymatic properties that correlate with differences in the metabolic needs of the rapidly dividing virulent tachyzoites *versus* the slowly replicating encysted bradyzoites [20], [36]. Furthermore, it appears that the presence of both TgENO1 and TgENO2 isoforms is not deleterious, as demonstrated by the simultaneous ectopic expression of these two isoenzymes in intracellular tachyzoites. Moreover, the absence of TgENO1 also leads to positive or negative changes in transcript levels of numerous target genes in tachyzoites or in bradyzoites. Thus, we propose a model for the functions of TgENO2 and TgENO1 in which they accumulate in the nucleus of actively dividing tachzyoites or bradyzoites and bind to gene promoters, leading to either repression or activation of transcription depending on other interacting partners, the promoter context, the intracellular niche, and differentiation status of the parasite. These novel nuclear functions of *T. gondii* enolases may involve changes in chromatin structure that control gene expression during parasite proliferation, virulence, differentiation, and cyst formation.

## Supporting Information

Figure S1 Nucleotide sequence of the putative promoter of TgMag1 (cyst matrix gene 1, TGME49_070240), a 787-bp region upstream of the start codon containing the double TTTCT-like motif TTTTTCTTCTC sequence (red). This region was sub-cloned into the reporter luciferase vector for promoter activity studies.
(PDF)

Figure S2 The first site-directed mutagenesis reaction of the putative TgMag1 promoter resulting in the disruption of a single TTTCT motif within the TTTTTCTTCTC motif of the promoter to *ATCGA*TCTC to give *Δ*_1_ *Tg*Mag1 plasmid, which was used in promoter activity assays.
(TIF)

Figure S3 The first mutagenized vector described above (*Δ*_1_ *Tg*Mag1) was subjected to a second round of mutagenesis to generate *ATCGAGCGC* (*Δ*_2_ *Tg*Mag2). This mutant promoter was also cloned upstream of the reporter luciferase construct for promoter activity assays.
(TIF)

Figure S4 Targeted deletion of the *ENO1* gene. A) Strategy for deleting the *TgENO1* gene in the Pru*Δku80Δhxgprt* strain using MPA selection. PCR1-4, locations of PCR products used to verify the genotype (not to scale; see Table S2). B) Validation of *TgENO1*-deleted clones based on the products of PCR1 (655 bp), PCR2 (356 bp), PCR3 (1,181 bp), and PCR4 (1,304 bp). Top panel: 12 randomly selected MPA-resistant clones were assayed by PCR1 and PCR2 (lanes 1–12). Lane 13 corresponds to parental Pru*Δku80* DNA assayed by PCR1 and PCR2. Lane 14, no-template control. Clones 1–12 exhibited perfect deletion of the *TgENO1* gene. Bottom panel: clones 1–6 with deletion of *TgENO1* were assayed by PCR4 (lanes 1–6) and PCR3 (lanes 11–16). Parental Pru*Δku80* DNA was also assayed by PCR4 (lane 7) and PCR3 (lane 17). Lanes 8 and 18 show no-template controls. DNA size ladder is shown in lanes 9 and 15. Clones 1–6 were validated as *TgENO1* knockouts with the genotype Pru*Δku80ΔTgeno1*. C) One knockout mutant was checked for perfect allelic integration and double homologous recombination using two primers (forward and reverse) specific for the open reading frame of TgENO1 and two other primers in the ORF (reverse) and the *TgENO1* promoter (forward). Superoxide dismutase (SOD) was used as a PCR control. The sequences of the primers are indicated in Table S3. All *in vitro* and *in vivo* phenotypic studies were performed using this *TgENO1* knockout mutant.
(TIF)

Table S1 **Primers used in this study.** The names and sequences of all primers used in this study are listed together with the associated gene targets and experimental applications. Underlined regions of primer sequences indicate an additional HA Tag, no gene-specific pLIC regions were required for either cloning. F =  forward primer, R =  reverse primer.
(DOC)

Table S2 Oligonucleotide primers used for construction of *ENO1* targeting vector.
(DOC)

Table S3 Oligonucleotide primers used for validation of *ENO1* deletion.
(DOC)

Table S4 Identification of ***T. gondii*** genes and promoters defined by genome-wide TgENO2 occupancy and ChIP-Seq. The list of gene targets was obtained using bioinformatics analyses and genome data from http://www.toxodb.org. After comparison of data from three independent experiments only genes that were identified in ChIP using anti-TgENO2 and anti-HA are shown. Genes that were non-specifically pulled down by the naïve sera used as a negative control were removed.
(XLS)

Table S5 Identification of ***T. gondii*** genes and ORFs defined by genome-wide TgENO2 occupancy and ChIP-Seq. The list of gene ORF targets was obtained using bioinformatics analyses and genome data from http://www.toxodb.org. After comparison of data from three independent experiments only genes that were identified in ChIP using anti-TgENO2 and anti-HA are shown.
(XLS)
