# Supplementary material for: Nuclear Glycolytic Enzyme Enolase of Toxoplasma gondii Functions as a Transcriptional Regulator
Source: PLoS One. 2014 Aug 25;9(8):e105820. doi: 10.1371/journal.pone.0105820 (PMC4143315; doi:10.1371/journal.pone.0105820)
Supplement: Figure S1 — Nucleotide sequence of the putative promoter of TgMag1 (cyst matrix gene 1, TGME49_070240), a 787-bp region upstream of the start codon containing the double TTTCT-like motif TTTTTCTTCTC sequence (red). This region was sub-cloned into the reporter luciferase vector for promoter activity studies. (PDF) [file pone.0105820.s001.pdf]

TgMag1

-787
GTTGCAGTTGCGTATGCCAGTGCGTCACAGCGCTGAACTACTGGCAGGTAGACACATCTGGCAAGCTACTGCTGTGTCTGAAGCGTGCCGATGTG

TGCGCGTACGCTTACAGAGAGCCTGCAAGACACTGGTTGGAAGACAAAATTTTTCTTCTCAAGAGTTGAGCTTTAGTTTGGTCACTCGCCGTTGG

TTGTTCTGTGTGCTAGACGTACTCTAACGCAAACCAGTCGAGGAACACACGAACGAGAGAGACGGCAATATCTCCCGTCGCGCTATCACACCGGG

TAAGGTCACGAGCTTCCGACTGGCACTGTGTATATCTTAATGGGGGACGCACGATTGGATCACCCAAATTCCGAGCGGTTTGAGGAAGGGAAACG

AGCGCGGTTAACAGCTCGTCGTTGGAGGACCTGTAGTGGTCGGGACTAAAGACGTATTAATGCAGTCGGACGCCACTGACCATCCATGGGGTTTA

GAGGCATGCTGATTTGCGGAGGAGGGGGGGAGGGTAAGCATCTTACCCGAAGATGATGGCACTCGGTCACTGAGAGACGATAGAGTGAACTGCG

GGGAAGGAGGTCGACTAGTGCGACCAGGGAAAGCACCTGGTAGGAAGAGCGTCGGGGGTGGCGGTAACAGGGGAGGCAACAGAAAGGCACAG

CAGCGCTACTTGGTGGGTGACGGCTATCACAGCGAAGGCGGCGCAGTGTTACACTACCGTAGTGCGTGTGGCACTCGTATTCACTGAAGTGATCG

-1
CTATTTTGTTTGCGTTACTGTGCACAGCAACATGGATTGCGGA

FIGURE S1
