# Supplementary figures and images for: Nuclear Glycolytic Enzyme Enolase of Toxoplasma gondii Functions as a Transcriptional Regulator
Source: PLoS One. 2014 Aug 25;9(8):e105820. doi: 10.1371/journal.pone.0105820 (PMC4143315; doi:10.1371/journal.pone.0105820)

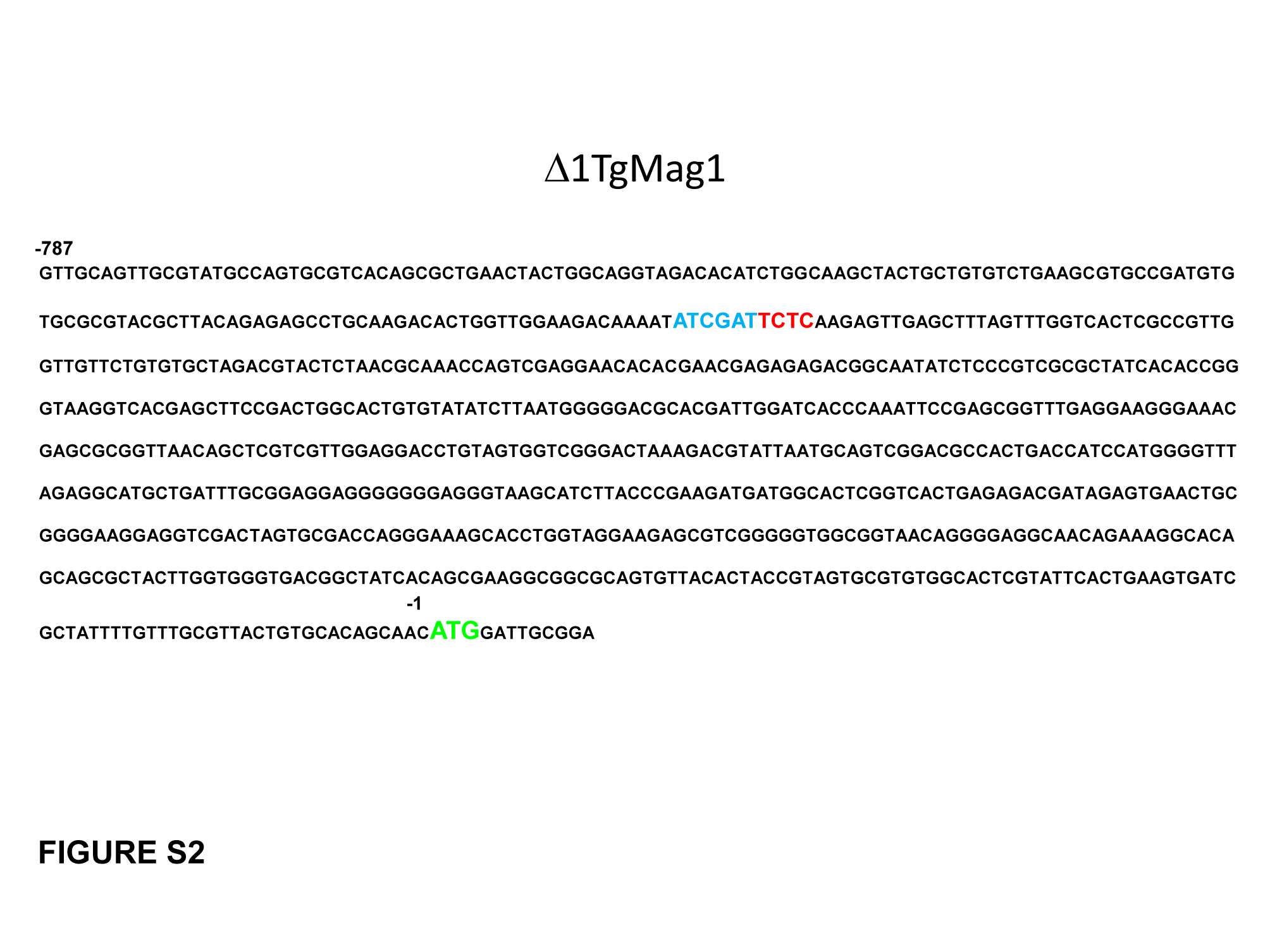

Supplement: Figure S2 — The first site-directed mutagenesis reaction of the putative TgMag1 promoter resulting in the disruption of a single TTTCT motif within the TTTTTCTTCTC motif of the promoter to ATCGATCTC to give Δ 1 TgMag1 plasmid, which was used in promoter activity assays. (TIF) [file pone.0105820.s002.tif]

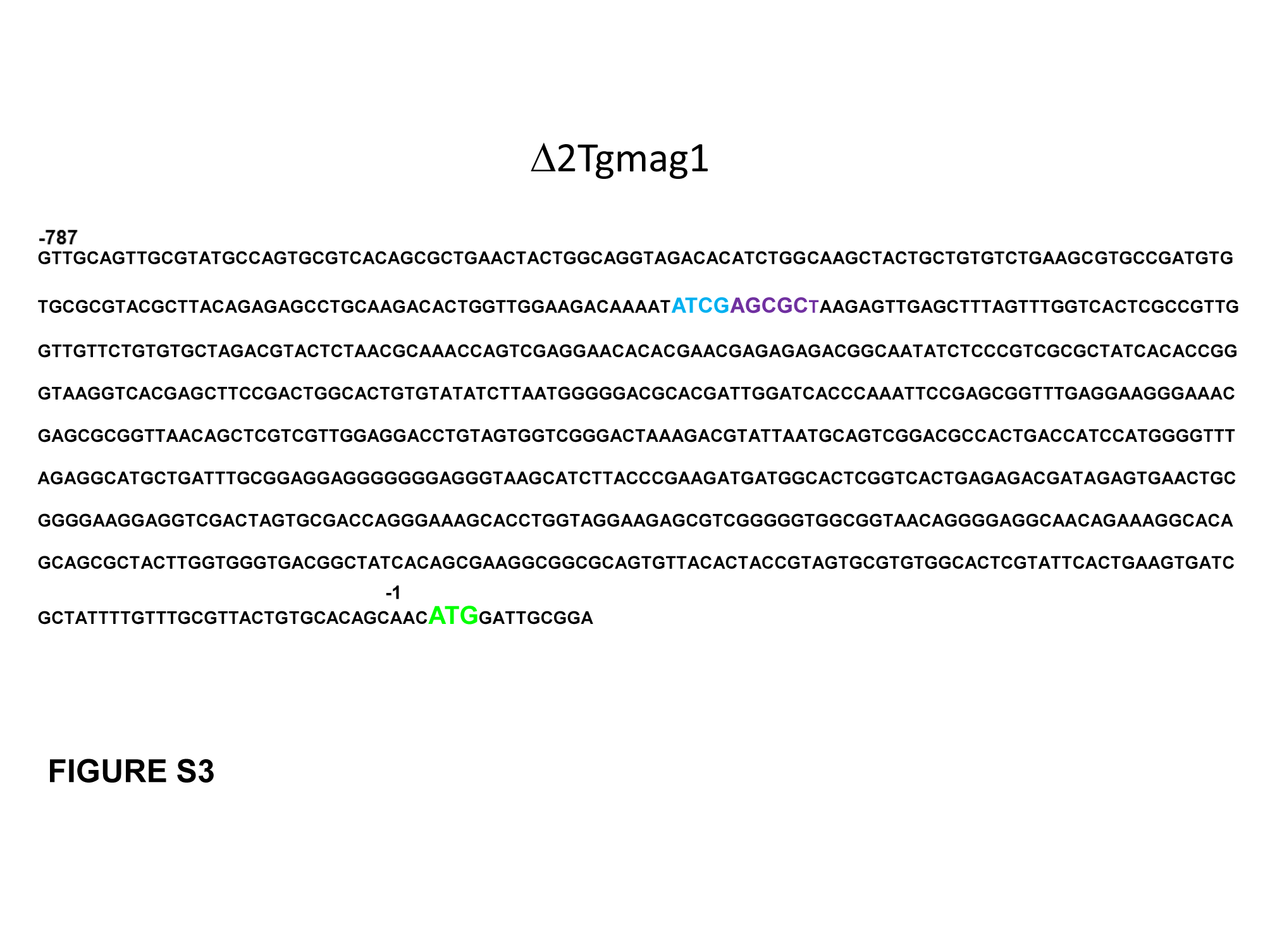

Supplement: Figure S3 — The first mutagenized vector described above (Δ 1 TgMag1) was subjected to a second round of mutagenesis to generate ATCGAGCGC (Δ 2 TgMag2). This mutant promoter was also cloned upstream of the reporter luciferase construct for promoter activity assays. (TIF) [file pone.0105820.s003.tif]

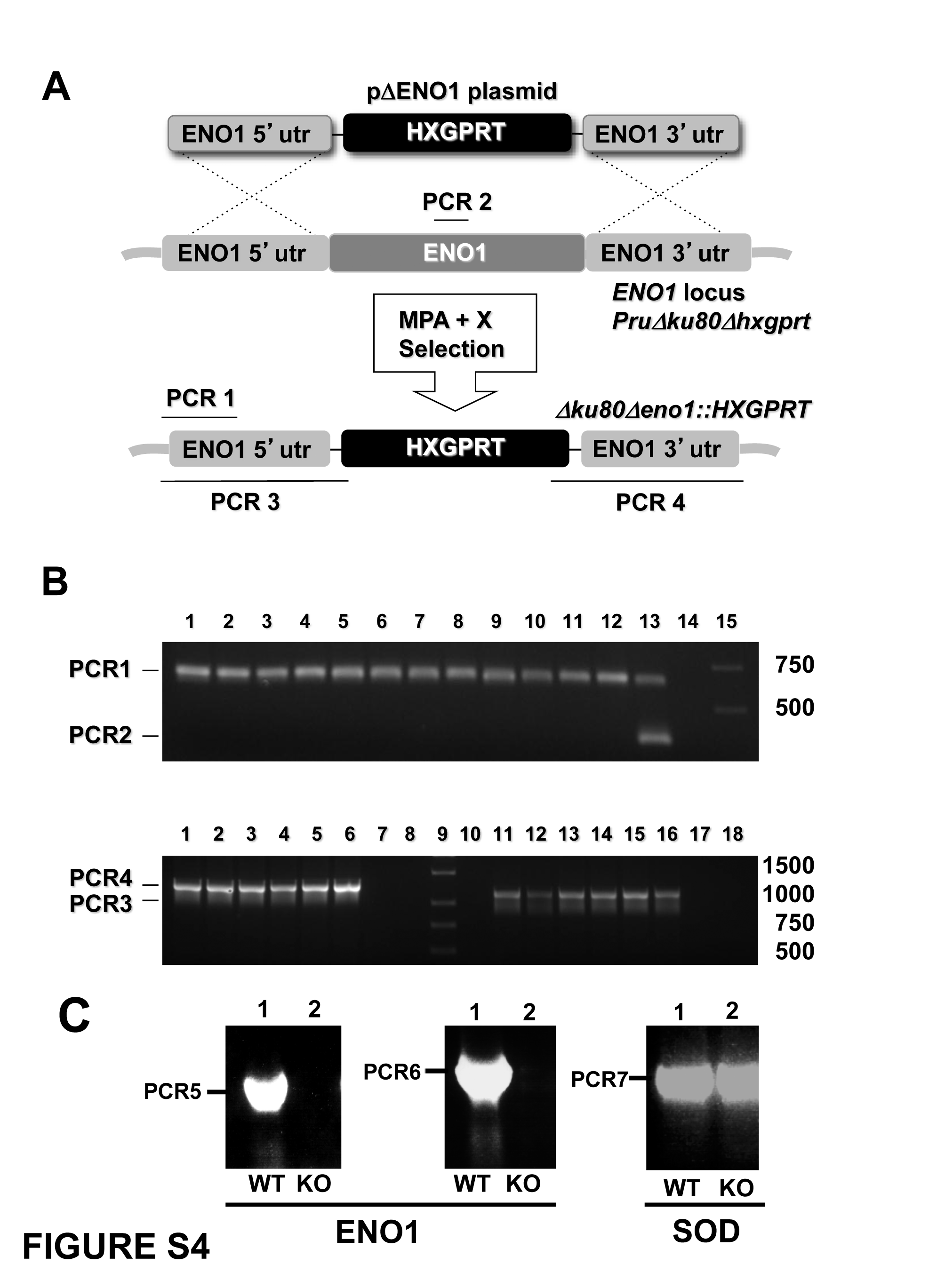

Supplement: Figure S4 — Targeted deletion of the ENO1 gene. A) Strategy for deleting the TgENO1 gene in the PruΔku80Δhxgprt strain using MPA selection. PCR1-4, locations of PCR products used to verify the genotype (not to scale; see Table S2). B) Validation of TgENO1-deleted clones based on the products of PCR1 (655 bp), PCR2 (356 bp), PCR3 (1,181 bp), and PCR4 (1,304 bp). Top panel: 12 randomly selected MPA-resistant clones were assayed by PCR1 and PCR2 (lanes 1–12). Lane 13 corresponds to parental PruΔku80 DNA assayed by PCR1 and PCR2. Lane 14, no-template control. Clones 1–12 exhibited perfect deletion of the TgENO1 gene. Bottom panel: clones 1–6 with deletion of TgENO1 were assayed by PCR4 (lanes 1–6) and PCR3 (lanes 11–16). Parental PruΔku80 DNA was also assayed by PCR4 (lane 7) and PCR3 (lane 17). Lanes 8 and 18 show no-template controls. DNA size ladder is shown in lanes 9 and 15. Clones 1–6 were validated as TgENO1 knockouts with the genotype PruΔku80ΔTgeno1. C) One knockout mutant was checked for perfect allelic integration and double homologous recombination using two primers (forward and reverse) specific for the open reading frame of TgENO1 and two other primers in the ORF (reverse) and the TgENO1 promoter (forward). Superoxide dismutase (SOD) was used as a PCR control. The sequences of the primers are indicated in Table S3. All in vitro and in vivo phenotypic studies were performed using this TgENO1 knockout mutant. (TIF) [file pone.0105820.s004.tif]
