# Supplementary material for: Nuclear Glycolytic Enzyme Enolase of Toxoplasma gondii Functions as a Transcriptional Regulator
Source: PLoS One. 2014 Aug 25;9(8):e105820. doi: 10.1371/journal.pone.0105820 (PMC4143315; doi:10.1371/journal.pone.0105820)
Supplement: Table S1 — Primers used in this study. The names and sequences of all primers used in this study are listed together with the associated gene targets and experimental applications. Underlined regions of primer sequences indicate an additional HA Tag, no gene-specific pLIC regions were required for either cloning. F = forward primer, R = reverse primer. (DOC) [file pone.0105820.s005.doc]

| **Primer name** | **Sequence** | | | **Used for** | **Comments** |
| --- | --- | --- | --- | --- | --- |
| ENO1-HA_F | GGATGCATATGGTGGTTATCAAGGACATCGTTGCA | | | Ectopic expression | pTUB5-Bleo vector |
| ENO1-HA_R | GGTTAATTAATCA**GAGGCTAGCGTAATCCGGAACATCGTATGGGTA**TTTTGGGTGTCGAAAGCTCTCTCCCGC | | | Ectopic expression | pTUB5-Bleo vector |
| ENO2-HA_F | GGCCATGCATATGGTGGCCATCAAGGACATCACTGCT | | | Ectopic expression | pSAG1-Bleo vector |
| ENO2-HA_R | GGCCTTAATTAATCA**GAGGCTAGCGTAATCCGGAACATCGTATGGGTA**GTTGGGATGGCGGAAGCCAGCGCCGGC | | | Ectopic expression | pSAG1-Bleo vector |
| HA-Tag sequence | **GAGGCTAGCGTAATCCGGAACATCGTATGGGTA** | | | HA-Tag |  |
|  |  |  |  | | |
| E2S2_F | TGATATAGTGAGACTCCTAAG | | | PCR | Enolase 2 promoter (TGME49_068850) |
| E2AS3_R | CCTTACACAATCGGTAAATCAGAA | | | PCR | Enolase 2 promoter (TGME49_068850) |
| ENO2-0-200_F | ACAGGCGTTGAACACCCAATTTTC | | | Q-PCR | Enolase 2 promoter (TGME49_068850) |
| ENO2-0-200_R | TGGCGTCAGGTGCGATAAGAAG | | | Q-PCR | Enolase 2 promoter (TGME49_068850) |
| ENO2-400-600_F | CACTACAACTGCTGCGAGTGAAAC | | | Q-PCR | Enolase 2 promoter (TGME49_068850) |
| ENO2-400-600_R | AAACCACACGACGAGGCTACC | | | Q-PCR | Enolase 2 promoter (TGME49_068850) |

| ENO1-600-800_F | CATGTGAGTTGCTCTGCGAGTTG | Q-PCR | Enolase 1 promoter (TGME49_068860) |
| --- | --- | --- | --- |
| ENO1-600-800_R | AACCACTGGTCTCTACGGCTTG | Q-PCR | Enolase 1 promoter (TGME49_068860) |
| ENO1-1000-1200_F | TGCCACGCTTTCTGTCGGATG | Q-PCR | Enolase 1 promoter (TGME49_068860) |
| ENO1-1000-1200_R | AAACTTGCCCTTCCTCGCTCTC | Q-PCR | Enolase 1 promoter (TGME49_068860) |
|  |  |  |  |
| PROM-083780_F | CAACAGCAGCACCACAGTTT | Q-PCR | Glucose-6-phosphate isomerase (TGME49_083780) |
| PROM-083780_R | CGTGTGTCTGCTGTGTCCTT | Q-PCR | Glucose-6-phosphate isomerase (TGME49_083780) |
| PROM-070250_F | AGAGCCTGCAAGACACTGGT | Q-PCR | GRA1  (TGME49_070250) |
| PROM-070250_R | GAATTTGGGTGATCCAATCG | Q-PCR | GRA1  (TGME49_070250) |
| PROM-070240_F | TGCAAGACACTGGTTGGAAG | Q-PCR | Cyst wall protein  (TGME49_070240) |
| PROM-070240_R | TACACAGTGCCAGTCGGAAG | Q-PCR | Cyst wall protein (TGME49_070240) |
| PROM-065450_F | AATTCGCAGAAAATCCATCG | Q-PCR | Hexokinase (TGME49_065450) |
| PROM-065450_R | GGCACACAGCTACGTCAGAA | Q-PCR | Hexokinase (TGME49_065450) |
| PROM-014320_F | TGCGACCAGCAAAGAGTATG | Q-PCR | Facilitative glucose transporter (TGME49_014320) |
| PROM-014320_R | TTGGTGCTAAATGCAAGCAG | Q-PCR | Facilitative glucose transporter (TGME49_014320) |
| PROM-005000_F | AGGAACTGAGCGAGACGGTA | Q-PCR | Phosphoglycerate mutase domain-containing protein (TGME49_005000) |
| PROM-005000_R | GTGTGTGGGAGTTGCCTTTT | Q-PCR | Phosphoglycerate mutase domain-containing protein (TGME49_005000) |
| PROM-062730_F | ACTTGGAGTCATCGCTGCTT | Q-PCR | Rhoptry kinase family protein ROP16 (TGME49_062730) |
| PROM-062730_R | TCGTCCTAGCGTCTGGTCTT | Q-PCR | Rhoptry kinase family protein ROP16 (TGME49_062730) |
| PROM-094200_F | GAGCATGCCAGTGTTCTTGA | Q-PCR | Glucose-6-phosphate dehydrogenase (TGME49_094200) |
| PROM-094200_R | GGGAGAGACAGACCAGTTGC | Q-PCR | Glucose-6-phosphate dehydrogenase (TGME49_094200) |
| PROM-022080_F | TGTGGAAAGCGTCTGAAGTG | Q-PCR | Hypothetical protein (TGME49_022080) |
| PROM-022080_R | TTCACGCTAGATCACGCAAG | Q-PCR | Hypothetical protein (TGME49_022080) |
|  |  |  |  |
| ORFA2_F | CTGCACACATCCAACAGCTT | Q-RT PCR | Glucose-6-phosphate isomerase (TGME49_083780) |
| ORFA2_R | TCAGCTTGATCGCTCTCTGA | Q-RT PCR | Glucose-6-phosphate isomerase (TGME49_083780) |
| ORF-070250_F2 | CTGGAGATGATGGGGAACAC | Q-RT PCR | GRA1 (TGME49_070250) |
| ORF-070250_R | GCTCCGAATTAAGCCCTTCT | Q-RT PCR | GRA1 (TGME49_070250) |
| ORFC1_F | GGCGTCCTTGTCTTCATTGT | Q-RT PCR | Cyst matrix protein (TGME49_070240) |
| ORFC1_R | TCGTCAACACGTCTTTCGAG | Q-RT PCR | Cyst matrix protein (TGME49_070240) |
| ORF-065450_F | GAGAACCCGGATCGTGTAGA | Q-RT PCR | Hexokinase (TGME49_065450) |
| ORF-065450_R | ACCCGTGTAGCCATAGTTGC | Q-RT PCR | Hexokinase (TGME49_065450) |
| ORF-014320_F | GGATATCCCACCCGAATCTT | Q-RT PCR | Facilitative glucose transporter (TGME49_014320) |
| ORF-014320_R | CCATCCGAAGTCGAGGATAA | Q-RT PCR | Facilitative glucose transporter (TGME49_014320) |
| ORF-005000_F | CATCCTTCCTCTGCTTTTCG | Q-RT PCR | Phosphoglycerate mutase domain-containing protein (TGME49_005000) |
| ORF-005000_R | GACCGTCGTCTGTCCTGAAT | Q-RT PCR | Phosphoglycerate mutase domain-containing protein (TGME49_005000) |
| ORF-062730_F | GTTTGAGGAAGCGCAAAAAG | Q-RT PCR | Rhoptry kinase family protein ROP16 (TGME49_062730) |
| ORF-062730_R | GCACCGAGACCTACTTCAGC | Q-RT PCR | Rhoptry kinase family protein ROP16 (TGME49_062730) |
| ORF-094200_F | CCGGAGTCGTTTCTCATTGT | Q-RT PCR | Glucose-6-phosphate dehydrogenase (TGME49_094200) |
| ORF-094200_R | GATCCCGTGGTGTAGCTCAT | Q-RT PCR | Glucose-6-phosphate dehydrogenase (TGME49_094200) |
| TbTGME49066960_F | TCCTCGCTCCTTTTGATGTC | Q-RT PCR | Tubulin beta chain (TGME49_066960) |
| TbTGME49066960_R | ATTGGAGACAATCCCGTCAG | Q-RT PCR | Tubulin beta chain (TGME49_066960) |

Mag-Luc-F : CCGGAAGCTTGTTGCAGTTGCGTATGCCAGTGCGT PCR Luciferase vector

Mag-Luc-R : CCGGAGATCTGTTGCTGTGCACAGTAACGCAAACAAAATAGC PCR Luciferase vector

**Supplementary Table S1**
