# Supplementary material for: Nuclear Glycolytic Enzyme Enolase of Toxoplasma gondii Functions as a Transcriptional Regulator
Source: PLoS One. 2014 Aug 25;9(8):e105820. doi: 10.1371/journal.pone.0105820 (PMC4143315; doi:10.1371/journal.pone.0105820)
Supplement: Table S2 — Oligonucleotide primers used for construction of ENO1 targeting vector. (DOC) [file pone.0105820.s006.doc]

**Supplementary Table S2. Oligonucleotide Primers used for Validation of *ENO1* deletion**

**Primer name Sequence Primer Use**

**ENO1CXF CGGAGTTTCGACTACCCAACTGC PCR1 and PCR3**

**ENO1EXR TGGAGCTCCGCATCTCGAAAGC PCR1**

**ENO1DF GACGAGTTCACTCATCTACGCGC PCR2**

**ENO1DR CGCGACCCAAGGTCCATCCT PCR2**

**ENO1CXR TCGCATGTAGTCTGCCCCTGTC PCR4**

**5’DHFRCXR GTTGGCCTACGTGACTTGCTGATG PCR3**

**3’DHFRCXF ACTGCGAACAGCAGCAAGATCG PCR4**

**5’EN01 ORF_F** **ATGGTGGTTATCAAGGACATCGTTGCA PCR5**

**3’ENO1 ORF_R** **TCACCGGATCTATGGAAAAC PCR5**

**5’ENO1EXT_F GCAACAAGCTGCTGTGTGCCACGC PCR6**

**3’ENO1EXT_R** **TCACCGGATCTATGGAAAAC PCR6**

**5’SOD_F GTTAATCGCCATGGTATTCAC PCR7**

**3’SOD_R ATTATTGAAATGCTCCAGTAG PCR7**
