# Supplementary material for: Nuclear Glycolytic Enzyme Enolase of Toxoplasma gondii Functions as a Transcriptional Regulator
Source: PLoS One. 2014 Aug 25;9(8):e105820. doi: 10.1371/journal.pone.0105820 (PMC4143315; doi:10.1371/journal.pone.0105820)
Supplement: Table S3 — Oligonucleotide primers used for validation of ENO1 deletion. (DOC) [file pone.0105820.s007.doc]

**Supplementary Table S3. Oligonucleotide primers used for construction of *ENO1* targeting vector**

**Primer name Sequence Primer Use**

**PMiniHXF** GATAAGCTTGATCAGCACGAAACCTTG HXGPRT cassette forward primer

**PMiniHXR** CCGCTCTAGAACTAGTGGATCCC HXGPRT cassette reverse primer

**ENO1F1** TTGGGTAACGCCAGGGTTTTCCCAGTCACGACGCCCGGG**GAAGGAGCACTTTCGCAGGTGC** PRU ENO1 KO 5’ target forward primer

**ENO1R1** GCGGGTTTGAATGCAAGGTTTCGTGCTGATCAAGTTTAAAC**CAGCGTACGTCCAACACGCAC** PRU ENO1 KO 5’ target reverse primer

**ENO1F2** TTCTGGCAGGCTACAGTGACACCGCGGTGGAGGGTTTAAAC**CGCCAATGTCCACACTCCTGC** PRU ENO1 KO 3’ target forward primer

**ENO1R2** GTGAGCGGATAACAATTTCACACAGGAAACAGCGCGGCCGC**ATGTCAGGAGTGAGTTGGAGAGCG** PRU ENO1 KO 3’ target reverse primer
